# Development of Resistance to Eravacycline by Klebsiella pneumoniae and Collateral Sensitivity-Guided Design of Combination Therapies

**DOI:** 10.1128/spectrum.01390-22

**Published:** 2022-08-16

**Authors:** Congjuan Xu, Xiaoya Wei, Yongxin Jin, Fang Bai, Zhihui Cheng, Shuiping Chen, Xiaolei Pan, Weihui Wu

**Affiliations:** a State Key Laboratory of Medicinal Chemical Biology, Key Laboratory of Molecular Microbiology and Technology of the Ministry of Education, Department of Microbiology, College of Life Sciences, Nankai University, Tianjin, China; b Department of Laboratory Medicine, 5th Medical Center of PLA General Hospital, Beijing, China; South China Sea Institute of Oceanology

**Keywords:** collateral sensitivity, eravacycline, *Klebsiella pneumoniae*, lon

## Abstract

The evolution of bacterial antibiotic resistance is exhausting the list of currently used antibiotics and endangers those in the pipeline. The combination of antibiotics is a promising strategy that may suppress resistance development and/or achieve synergistic therapeutic effects. Eravacycline is a newly approved antibiotic that is effective against a variety of multidrug-resistant (MDR) pathogens. However, the evolution of resistance to eravacycline and strategies to suppress the evolution remain unexplored. Here, we demonstrated that a carbapenem-resistant Klebsiella pneumoniae clinical isolate quickly developed resistance to eravacycline, which is mainly caused by mutations in the gene encoding the Lon protease. The evolved resistant mutants display collateral sensitivities to β-lactam/β-lactamase inhibitor (BLBLI) combinations aztreonam/avibactam and ceftazidime-avibactam. Proteomic analysis revealed upregulation of the multidrug efflux system AcrA-AcrB-TolC and porin proteins OmpA and OmpU, which contributed to the increased resistance to eravacycline and susceptibility to BLBLIs, respectively. The combination of eravacycline with aztreonam/avibactam or ceftazidime-avibactam suppresses resistance development. We further demonstrated that eravacycline-resistant mutants evolved from an NDM-1-containing K. pneumoniae strain display collateral sensitivity to aztreonam/avibactam, and the combination of eravacycline with aztreonam/avibactam suppresses resistance development. In addition, the combination of eravacycline with aztreonam/avibactam or ceftazidime-avibactam displayed synergistic therapeutic effects in a murine cutaneous abscess model. Overall, our results revealed mechanisms of resistance to eravacycline and collateral sensitivities to BLBLIs and provided promising antibiotic combinations in the treatment of multidrug-resistant K. pneumoniae infections.

**IMPORTANCE** The increasing bacterial antibiotic resistance is a serious threat to global public health, which demands novel antimicrobial medicines and treatment strategies. Eravacycline is a newly approved antibiotic that belongs to the tetracycline antibiotics. Here, we found that a multidrug-resistant Klebsiella pneumoniae clinical isolate rapidly developed resistance to eravacycline and the evolved resistant mutants displayed collateral sensitivity to antibiotics aztreonam/avibactam and ceftazidime-avibactam. We demonstrated that the combination of eravacycline with aztreonam/avibactam or ceftazidime-avibactam repressed resistance development and improved the treatment efficacies. We also elucidated the mechanisms that contribute to the increased resistance to eravacycline and susceptibility to aztreonam/avibactam and ceftazidime-avibactam. This work demonstrated the mechanisms of antibiotic resistance and collateral sensitivity and provided a new therapeutically option for effective antibiotic combinations.

## INTRODUCTION

Klebsiella pneumoniae is a Gram-negative opportunistic pathogen that causes various nosocomial infections, such as urinary tract infection, bacteremia, pneumonia, liver abscess, etc. (1). Multidrug-resistant (MDR) K. pneumoniae isolates, particularly those resistant to carbapenem antibiotics (CRKP), have been increasingly reported and become a serious public health threat since the early 2000s (2, 3). Colistin is considered one of the last-line treatment options for infections caused by CRKP (4). However, *mcr-1*-producing colistin-resistant K. pneumoniae strains have been reported in countries around the world (5). Therefore, new antibiotics and strategies to retard resistance development are in urgent need.

Eravacycline is a novel synthetic fluorocycline that was approved by the USA Food and Drug Administration (FDA) in 2018 for the treatment of complicated intra-abdominal infections (6). Eravacycline has shown potent *in vitro* activity against most Gram-positive and Gram-negative pathogens, including carbapenem-resistant Enterobacteriales (CRE) (7). Although it has a structure and action mechanisms similar to those of tigecycline, eravacycline has been reported to be 2 to 4-fold more active than tigecycline against common clinical Gram-positive and Gram-negative aerobic bacterial species (8). Therefore, eravacycline is likely to be used in the treatment of infections caused by CRKP.

*In vitro* evolution experiments in Acinetobacter baumannii demonstrated that overexpression of the efflux pump genes *adeABC* results in eravacycline resistance (9). In Enterococcus faecalis, overexpression of a BMP family ABC transporter substrate-binding protein gene *RS00630* increased the frequency of eravacycline heteroresistance (10). However, the resistance mechanism of eravacycline in K. pneumoniae, especially CRKP has not been elucidated in detail.

Collateral sensitivity-guided antibiotic combinations and cycling have been proposed to repress the development of resistance and achieve effective treatment (11, 12). Collateral sensitivity is the phenomenon in which the development of resistance to one antibiotic can result in increased susceptibility to other antibiotics (11–13). For instance, a previous study demonstrated that tigecycline-resistant E. coli mutants were more susceptible to nitrofurantoin, which was due to the increased drug uptake and upregulation of nitroreductase enzymes (12).

Here, we studied the evolution and mechanisms responsible for the resistance to eravacycline of CRKP strains through *in vitro* passaging experiments. Our results revealed that mutations in the *lon* gene increased bacterial resistance to eravacycline. Based on the collateral sensitivities of the evolved eravacycline-resistant mutants, we found that the combination of eravacycline with aztreonam/avibactam or ceftazidime-avibactam retarded the development of resistance of CRKP *in vitro*. In addition, the combinations showed synergistic bactericidal activity in a murine subcutaneous abscess model. Overall, our results provide a strategy to increase the treatment efficacy of eravacycline while suppressing the development of resistance.

## RESULTS

### Characterization of the MDR clinical isolate Kp43.

A K. pneumoniae clinical isolate Kp43 was resistant to a variety of antibiotics (Table 1). Whole-genome sequencing (WGS) and multilocus sequence typing (MLST) analysis (https://bigsdb.Pasteur.fr/klebsiella) revealed that Kp43 belonged to ST11, a dominant clone that accounts for up to 60% of carbapenem-resistant K. pneumoniae in China (14). Kp43 contained a 5.48 Mbp chromosome and three plasmids, designated pKp43_1 (277929 bp), pKp43_2 (138350 bp), and pKp43_3 (114288 bp) (Table S1 in Supplemental File 1). Sequence analysis by ResFinder 4.1 revealed that Kp43 harbored seven β-lactamase genes, including *blaSHV-11* (chromosome), *blaOXA-1* (pKp43_1), *blaLAP-2* (pKp43_2), *blaSHV-12* (pKp43_2), *blaCTX-M-65* (pKp43_3), *blaKPC-2* (pKp43_3), and *blaTEM-1B* (pKp43_3). In addition, Kp43 possessed a variety of genes that were involved in resistance to aminoglycoside, quinolone, macrolide, and tetracycline antibiotics (Table S2 in Supplemental File 1). However, Kp43 was susceptible to the BLBLI combinations ceftazidime-avibactam and aztreonam/avibactam (Table 1) (15, 16), presumably due to the ability of avibactam to inhibit all the β-lactamases carried by Kp43. Meanwhile, the MIC of eravacycline was 2 mg/L for Kp43 (Table 1). Although the breakpoint for eravacycline has not been established, K. pneumoniae isolates with a MIC ≤ 2 mg/L were considered to be susceptible (17, 18).

**TABLE 1:** MICs (mg/L) of the K. pneumoniae clinical isolates

Strains	ERV^*a*^	TGC	TC	CAZ	CAZ /AVI^*b*^^,^^*c*^	ATM	ATM /AVI^*b*^^,^^*c*^	MEM	IPM	AZM	TOB	GM	CIP	RIF	FOS
Kp43^*d*^	2	2	>512	256	2	>512	1	128	64	512	>512	>512	512	256	>512
Kp17^*d*^	0.25	0.25	ND	>64	>64	0.125	0.125	8	8	>64	ND	>64	64	ND	ND

a ERV, eravacycline; TGC, tigecycline; TC, tetracycline; CAZ, ceftazidime; AVI, avibactam; ATM, aztreonam; MEM, meropenem; IPM, imipenem; AZM, azithromycin; TOB, tobramycin; GM, gentamicin; CIP, ciprofloxacin; RIF, rifampicin; FOS, fosfomycin; ND: not determined.

b Avibactam was fixed at 4 mg/L.

c Breakpoints for ceftazidime, ≤8 mg/L and aztreonam, ≤4 mg/L (13, 14).

d Data represent results from three independent experiments.

### Mutations in the *lon* gene contributed to bacterial resistance to eravacycline.

**FIG 1:**
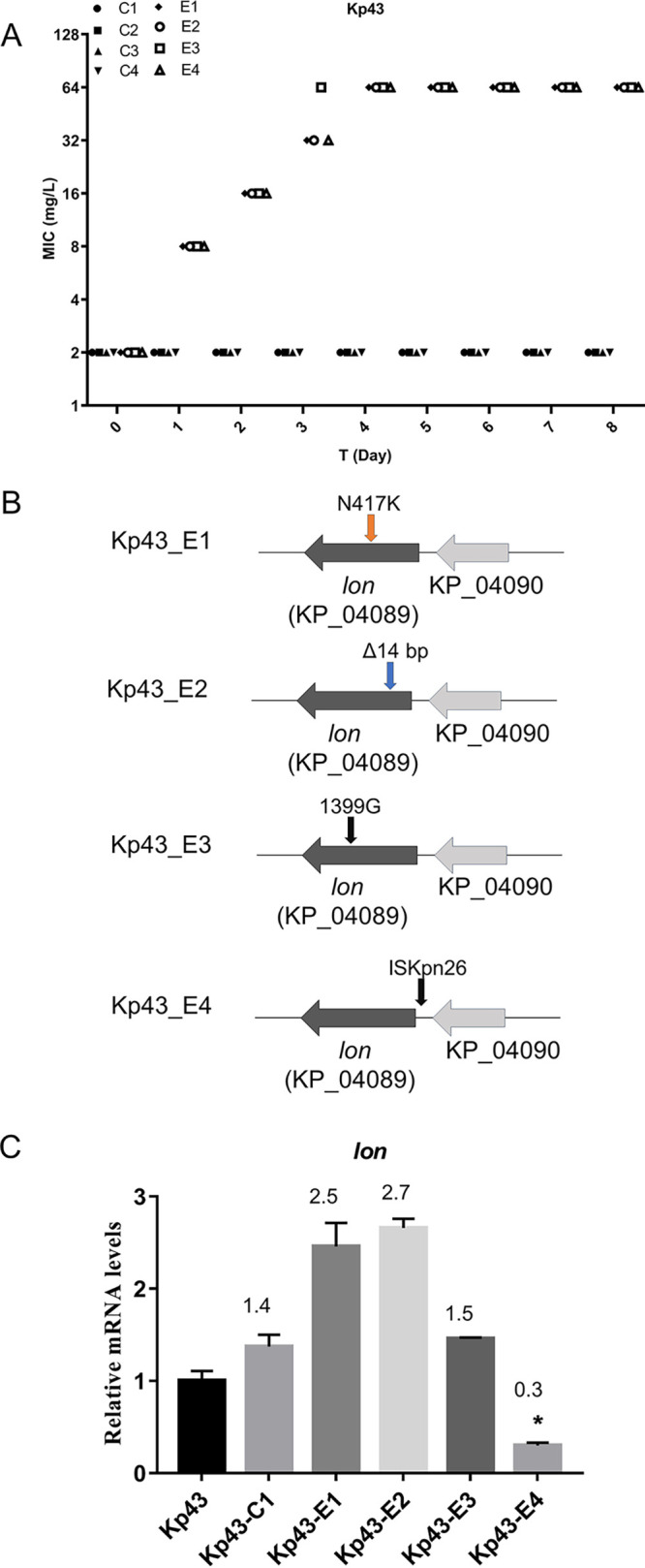
Development of eravacycline resistance by Kp43. (A) Dynamics of stepwise resistance development to eravacycline in Kp43. Four parallel repeats were performed in the presence of eravacycline (designated E1 to E4). Another four parallel repeats were passaged in LB (designated C1 to C4). (B) Schematic presentation of mutations in the *lon* gene in eravacycline-resistant mutants. (C) Relative mRNA levels of the *lon* gene. The relative mRNA levels of the indicated genes were determined by real-time PCR using primers targeting the unmutated regions. The *rpoB* gene was used as an internal control. The numbers above the columns are the fold changes of the *lon* mRNA levels in the Kp43-C1 and E1 to E4 strains compared to that in the Kp43. *, *P* < 0.05, by Student's *t* test.

To examine the development of resistance of Kp43 to eravacycline, we performed an *in vitro* passage assay with four parallel repeats (Fig. 1A). We obtained one eravacycline-resistant strain from each of the repeats with a MIC of 64 mg/L, which was 32-fold of the initial MIC. The four strains were named Kp43-E1 to Kp43-E4, and the strains passaged in cation-adjusted Mueller-Hinton (CAMHB) were named Kp43-C1 to Kp43-C4.

WGS was performed to identify mutations in the genomes of the four resistant strains in comparison with Kp43-C1 and the parental strain Kp43. The four mutants contained either mutations in the *lon* gene coding region or a transposon insertion in the promoter (Fig. 1B, Table S3 in Supplemental File 1), which were confirmed by PCR and sequencing (unpublished data).

Lon is an ATP-dependent protease that is involved in biofilm formation, motility, pathogenicity, and stress responses (19). Kp43 was resistant to most antibiotics normally used in cloning but remained susceptible to apramycin. Thus, we used a vector with apramycin as the selection marker for complementation (Table S4 in Supplemental File 1). Complementation with a wild-type *lon* gene reduced the MICs for Kp43-E1, Kp43-E2, and Kp43-E3 by 2-fold, whereas the MIC for Kp43-E4 was restored to the level of the parental strain Kp43 (Table 2). In Kp43-E4, an insertion sequence (ISKpn26) was inserted upstream of the coding region of the *lon* gene. A quantitative real-time (qRT)-PCR assay revealed that the insertion reduced the expression level of *lon* (Fig. 1C). Kp43-E1 contained a C1251A mutation in the coding region of *lon*, which altered the 417th asparagine to lysine. Kp43-E2 and Kp43-E3 contained a 14-bp deletion and an insertion of G at the 698th bp and 1339th bp of the coding region of *lon*, respectively (Fig. 1B). Because Lon functions as a dodecamer through N-terminal portion-mediated assembly (20), we suspected that the mutated and truncated Lon proteins might bind to the complemented full-length Lon protein, which interfered with the function of the complex, thus resulting in partial restoration of the sensitivity. To further confirm the role of the *lon* mutations in the eravacycline resistance, we constructed a *lon* deletion mutant in an eravacycline-sensitive clinical isolate P4325. Deletion of the *lon* gene increased the MIC of eravacycline by 2-fold, which was restored to the wild-type level by complementation with the wild-type *lon* gene. However, complemented with the mutant *lon* genes from Kp43-E1, Kp43-E2, Kp43-E3, and Kp43-E4 did not reduce the MIC (Table 3). In combination, these results demonstrated that mutations in the *lon* gene contributed to bacterial resistance to eravacycline.

**TABLE 2:** MICs (mg/L) of eravacycline for indicated K. pneumoniae strains

	Kp43/	Kp43-E1/	Kp43-E1/	Kp43-E2/	Kp43-E2/	Kp43-E3/	Kp43-E3/	Kp43-E4/	Kp43-E4/
Strains^*a*^	pKP^*b*^	pKP	pKP-lonKp43	pKP	pKP-lonKp43	pKP	pKP-lonKp43	pKP	pKP-lonKp43
MIC	2	32	16	32	16	32	16	32	2

a Data represent results from three independent experiments.

b Strains containing the pKp or pKP-lonKp43 plasmid were cultured at 30°C.

**TABLE 3:** MICs (mg/L) of indicated K. pneumoniae strains

Strains^*a*^	ERV^*b*^	ATM/AVI^*b*^	CAZ/AVI^*b*^
P4325/pUCP24NP	0.25	0.03125	0.125
P4325Δlon/pUCP24NP	0.5	0.0157625	0.0625
P4325Δlon/pUCP24NP-lonP4325	0.25	0.03125	0.125
P4325Δlon/pUCP24NP-lonKp43-E1	0.5	0.0157625	0.0625
P4325Δlon/pUCP24NP-lonKp43-E2	0.5	0.0157625	0.0625
P4325Δlon/pUCP24NP-lonKp43-E3	0.5	0.0157625	0.0625
P4325Δlon/pUCP24NP-lonKp43-E4	0.5	0.0157625	0.0625

a Data represent results from three independent experiments.

b ERV, eravacycline; CAZ, ceftazidime; AVI, avibactam; ATM, aztreonam.

### Collateral sensitivities of the eravacycline-resistant strains.

The quick development of eravacycline resistance indicated an urgent demand for a strategy to repress resistance development before large-scale clinical use. The combination of antibiotics with collateral sensitivities is a promising strategy to suppress the development of antibiotic resistance (21, 22). We, thus, examined the antibiotic resistance profiles of the evolved eravacycline-resistant strains. All four strains were more resistant to tigecycline and azithromycin (Table 4). However, the MICs of ceftazidime-avibactam and aztreonam/avibactam were reduced by 2-fold (Table 4). Complementation with the *lon* gene in Kp43-E4 restored bacterial resistance to ceftazidime-avibactam and aztreonam/avibactam (Table 4). In addition, deletion of the *lon* gene in the P4325 strain reduced the MICs of ceftazidime-avibactam and aztreonam/avibactam by 2-fold, which was restored by complementation with a wild-type *lon* gene but not the mutant *lon* genes from Kp43-E1, Kp43-E2, Kp43-E3, and Kp43-E4 (Table 3). Collectively, these results demonstrated the role of *lon* in collateral sensitivity.

**TABLE 4:** MICs (mg/L) of the eravacycline-resistant strains

Strains^*a*^	ERV^*b*^	ERV + PaβN^*c*^	TGC^*b*^	AZM	TOB^*b*^	CIP^*b*^	ATM	ATM/AVI	CAZ^*b*^	CAZ/AVI^*b*^
Kp43^*c*^	2	0.25	2	512	>512	>512	>512	1	256	2
Kp43-C1	2	0.25	2	512	>512	>512	>512	1	256	2
Kp43-E1	64	4	64	>512	>512	>512	>512	0.5	256	1
Kp43-E2	64	2	64	>512	>512	>512	>512	0.5	256	1
Kp43-E3	64	2	64	>512	>512	>512	>512	0.5	256	1
Kp43-E4	64	2	64	>512	>512	>512	>512	0.5	256	1
Kp43/pKP	2	ND	2	512	>512	ND	>512	1	256	2
Kp43-E4/pKP	32	ND	32	>512	>512	ND	>512	0.5	256	1
Kp43-E4/pKP-lonKp43	2	ND	2	512	>512	ND	>512	1	256	2

a Data represent results from three independent experiments.

b ERV, eravacycline; TGC, tigecycline; AZM, azithromycin; TOB, tobramycin; CIP, ciprofloxacin; ATM, aztreonam; CAZ, ceftazidime; AVI, avibactam; ND, not determined.

c PAβN: the efflux pump inhibitor, final concentration is 100 mg/L.

### Mechanisms of the evolved resistance to eravacycline and collateral sensitivities to the BLBLI combinations.

**FIG 2:**
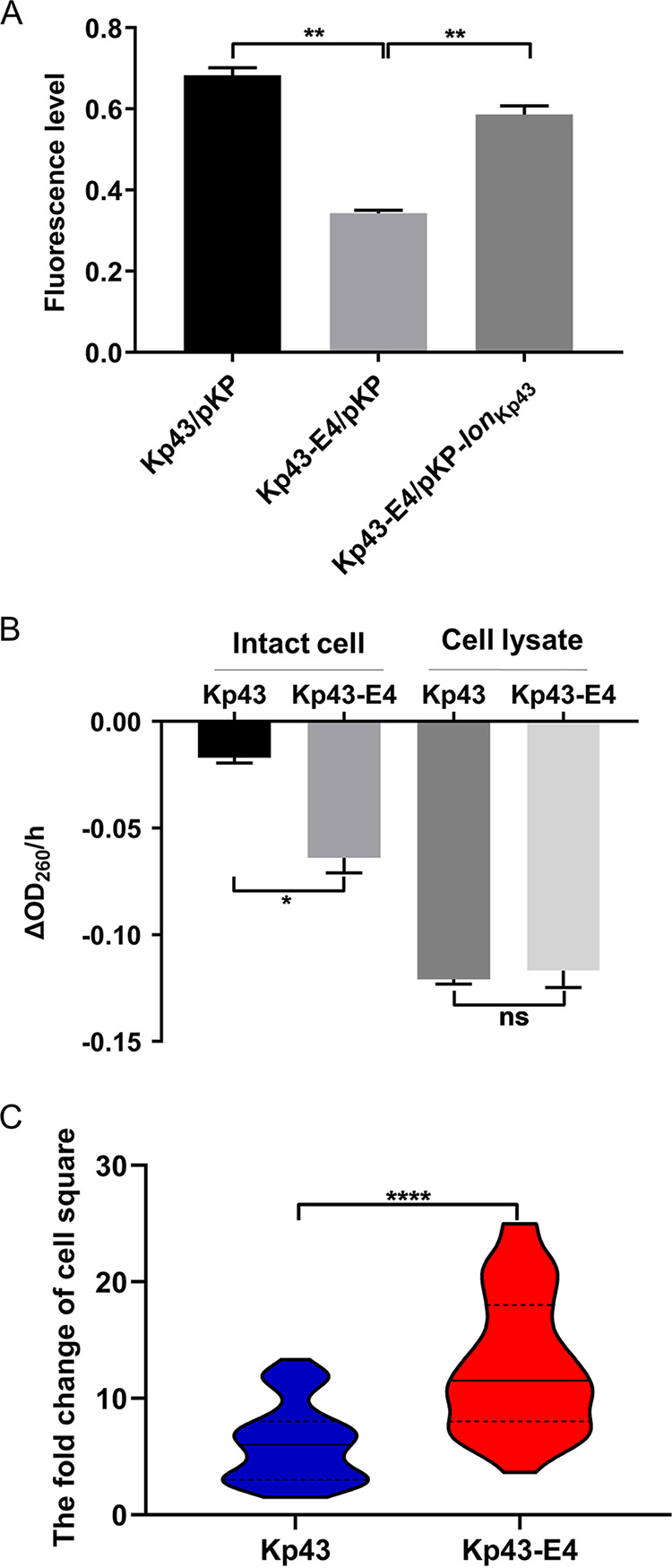
Intracellular accumulation of eravacycline and influx of ceftazidime. (A) Intracellular amounts of eravacycline in Kp43, Kp43-E4, and the complemented strain. The relative intracellular accumulation of eravacycline was determined by measuring the fluorescence (excitation at 400 nm and emission at 520 nm) after incubation with 50 mg/L eravacycline for 15 min. (B) Hydrolysis rates of ceftazidime in intact bacteria or bacterial lysates. *, *P* < 0.05; ns, not significant by Student's *t* test. (C) Violin plots displaying the fold change of cell square (length × width) before and after ceftazidime/avibactam (CAZ/AVI) treatment. The results were from 40 cells in four random fields. ****, *P* < 0.0001 by Student's *t* test.

Eravacycline and the BLBLI combinations inhibited distinct targets, cytoplasmic ribosome and periplasmic PBP proteins, respectively. Thus, we suspected that mutations of the *lon* gene might affect bacterial resistance by altering drug influxes. Indeed, the intracellular amount of eravacycline was reduced in Kp43-E4 cells, which was restored by complementation with a *lon* gene (Fig. 2A).

The influx rates of ceftazidime were measured by combining the influx of the drug and its subsequent hydrolysis by endogenous β-lactamases in intact bacterial cells as previously described (23). Compared to Kp43, Kp43-E4 caused faster hydrolysis of ceftazidime. As a control, cell lysates of Kp43 and Kp43-E4 resulted in similar hydrolysis rates, indicating similar expression levels of the β-lactamases (Fig. 2B). These results suggest an increased influx of ceftazidime in Kp43-E4. Consistently, Kp43-E4 displayed more severe swelling (increase in cell length and width) than Kp43 following ceftazidime-avibactam treatment (Fig. 2C, Fig. S1 in Supplemental File 1), indicating a higher periplasmic drug level in Kp43-E4.

**FIG 3:**
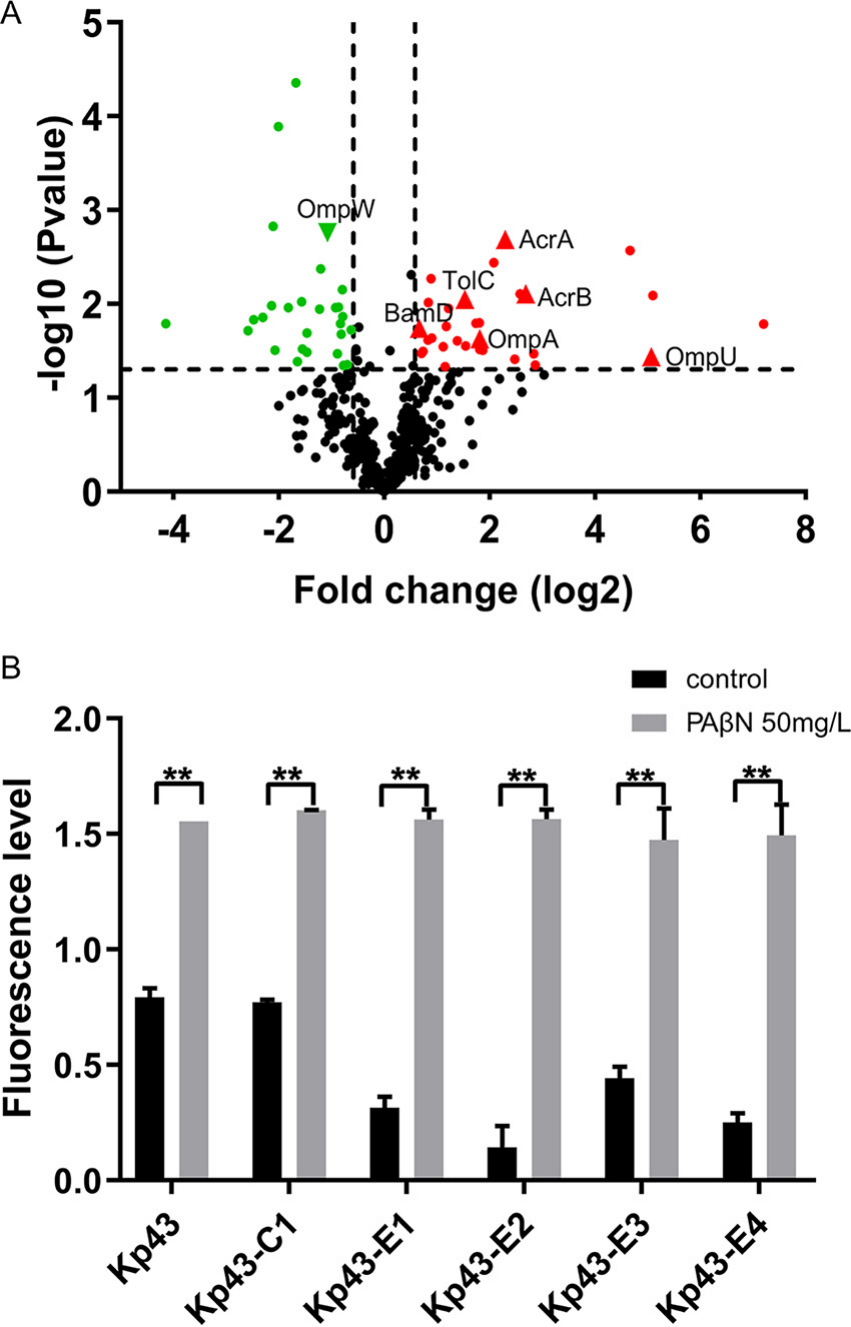
Membrane protein profiles and efflux of eravacycline. (A) Volcano plot depicting membrane proteins of Kp43 and Kp43-E4. The *x*-axis of the graph shows log_2_ changes in membrane proteins (Kp43-E4 versus Kp43). Red and green dots represent significantly upregulated and downregulated proteins, respectively. (B) Intracellular amounts of eravacycline. The relative intracellular accumulation of eravacycline was determined by measuring the fluorescence (excitation at 400 nm and emission at 520 nm) after incubation with 50 mg/L eravacycline for 15 min with or without PAβN pretreatment. **, *P* < 0.01, by Student's *t* test.

Because intracellular drug levels were largely determined by membrane permeability and efflux, we compared the membrane protein profiles between Kp43 and Kp43-E4. There were 31 and 29 proteins upregulated and downregulated, respectively, in Kp43-E4 (Fig. 3A and Table S5 in Supplemental File 1). Of note, the multidrug efflux system AcrA-AcrB-TolC, which has been shown to contribute to bacterial resistance to tetracycline antibiotics, was upregulated in Kp43-E4. Pretreatment with the efflux pump inhibitor phenylalanine-arginine β-naphthylamide (PAβN) resulted in similar intracellular amounts of eravacycline (Fig. 3B). In addition, the presence of PAβN reduced the MIC of eravacycline for Kp43 and the evolved resistant mutants by 8 and 16 to 32-fold, respectively, which reduced the difference between Kp43 and the mutants from 32-fold to 8 to 16-fold (Table 4).

The outer membrane proteins OmpA and OmpU were upregulated in Kp43-E4 (Fig. 3A), which might contribute to the increased susceptibility to aztreonam/avibactam and ceftazidime-avibactam. Overexpression of *ompA* in Kp43 did not affect the MICs. However, overexpression of *ompU* reduced the MIC of ceftazidime-avibactam by 2-fold (Table 5). Furthermore, co-overexpression of *ompA* and *ompU* reduced the MICs of both aztreonam/avibactam and ceftazidime-avibactam (Table 5). These results demonstrated that the upregulation of OmpA and OmpU increased bacterial susceptibility to BLBLIs, in which OmpU played a more important role.

**TABLE 5:** MICs (mg/L) of indicated K. pneumoniae strains

Strains^*a*^	ATM/AVI^*b*^	CAZ/AVI^*b*^
Kp43/pUCP24a	1	2
Kp43/pUCP24a-ompA	1	2
Kp43/pUCP24a-ompU	1	1
Kp43/pUCP24a-ompAU	0.5	1
Kp43-E4/pUCP24a	0.5	1

a Data represent results from three independent experiments.

b ATM, aztreonam; CAZ, ceftazidime; AVI, avibactam.

### The combination of eravacycline with aztreonam/avibactam or ceftazidime-avibactam suppressed resistance development.

**FIG 4:**
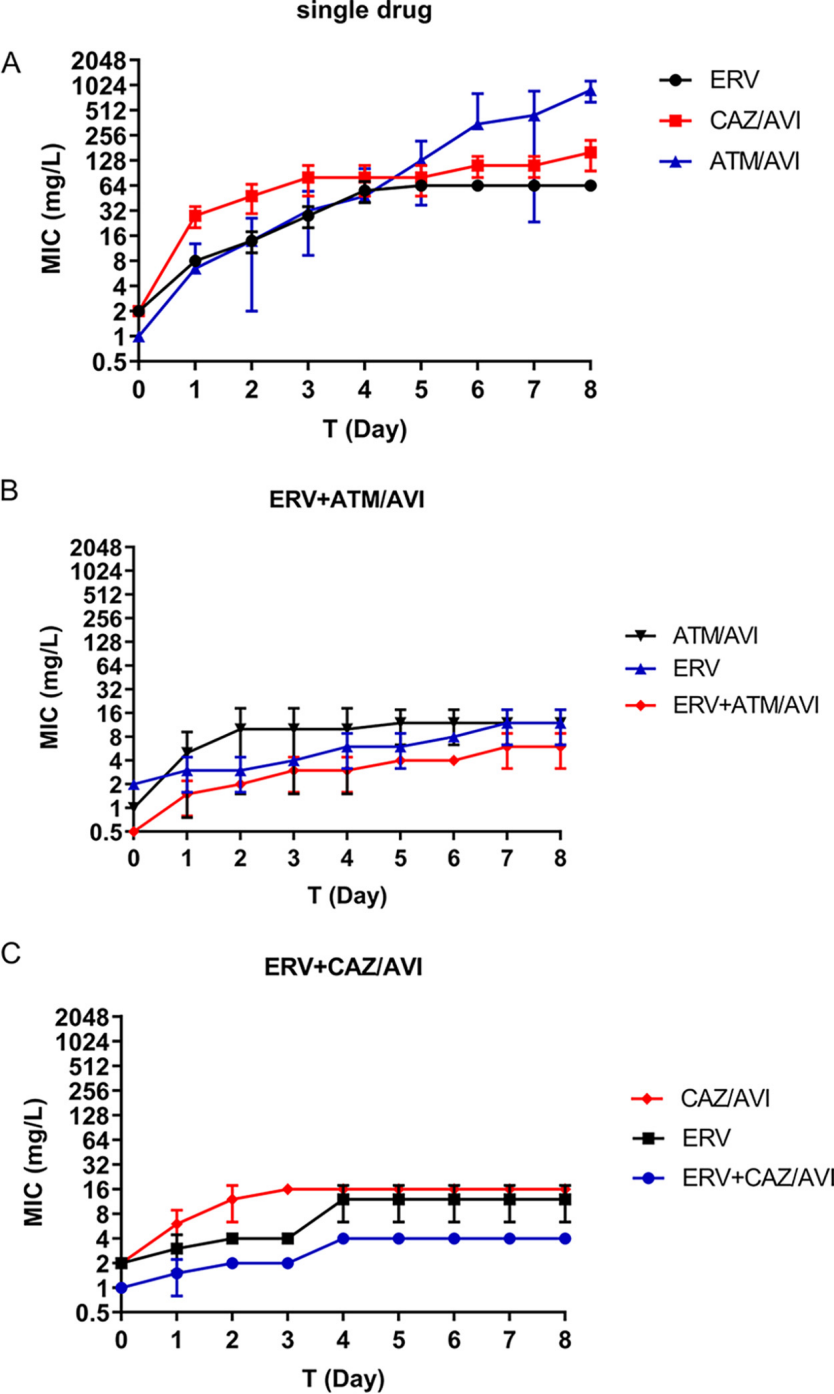
Effects of eravacycline in combination with aztreonam/avibactam or ceftazidime-avibactam on the development of antibiotic resistance in Kp43. (A) Passaging of Kp43 in eravacycline (ERV), aztreonam/avibactam (ATM/AVI), and ceftazidime-avibactam (CAZ/AVI) alone. MICs of the corresponding antibiotics were determined each day. (B, C) Passaging of Kp43 in ERV+ATM/AVI (1:1) (B) or ERV+CAZ/AVI (1:1) (C). MICs of the individual antibiotics and the corresponding combinations were determined each day. Error bars indicate SEM.

The collateral sensitivities of the eravacycline-resistant strains to ceftazidime-avibactam and aztreonam/avibactam indicated possible repression of resistance development for combination therapies. We then examined whether there was reciprocal collateral sensitivity between the antibiotics. Ceftazidime-avibactam- and aztreonam/avibactam-resistant mutants were obtained by *in vitro* passaging assays with four parallel repeats for each drug. Kp43 quickly evolved resistance to the two drugs (Fig. 4A). Resistant mutants from each of the passaging assays were isolated on day 8 (Table 6). Because it has been reported that ceftazidime-avibactam and aztreonam/avibactam resistance are related to Klebsiella pneumoniae carbapenemases (KPCs) mutation (24, 25), we sequenced the *KPC* genes in the evolved resistant mutants and found mutations (Table S6 in Supplemental File 1), However, expression of the mutated *KPC* genes in a ceftazidime-avibactam- and aztreonam/avibactam-sensitive strain PA4325 did not increase the resistance to ceftazidime-avibactam or aztreonam/avibactam, indicating that the mutations were not responsible for the resistance (Table S7 in Supplemental File 1). The MICs of eravacycline for the ceftazidime-avibactam-resistant strains decreased 2-fold, indicating collateral sensitivity. However, no change was observed in the aztreonam/avibactam-resistant mutants (Table 6).

**TABLE 6:** MICs (mg/L) of the BLBLI-resistant mutants

Strains^*a*^	ATM/AVI^*b*^	CAZ/AVI	ERV
Kp43-CA1	ND	128	1
Kp43-CA2	ND	256	1
Kp43-CA3	ND	128	1
Kp43-CA4	ND	128	1
Kp43-AA1	512	ND	2
Kp43-AA2	1024	ND	2
Kp43-AA3	1024	ND	2
Kp43-AA4	1024	ND	2

a Data represent results from three independent experiments.

b CA, ceftazidime-avibactam resistant strains; AA, aztreonam/avibactam resistant strains; ATM, aztreonam; CAZ, ceftazidime; AVI, avibactam; ERV, eravacycline; ND: not determined.

To examine the effects of the combination of eravacycline with aztreonam/avibactam or ceftazidime-avibactam on resistance development, we performed sequential passaging experiments of Kp43 with the combined antibiotics (26). In the presence of the eravacycline-aztreonam/avibactam combination, the MICs of eravacycline, aztreonam/avibactam, and the combined antibiotics increased 4, 8, and 8-fold, respectively, compared with the cells passaged in CAMHB (Fig. 4B). Similarly, in the presence of the eravacycline-ceftazidime-avibactam combination, the MICs of eravacycline, ceftazidime-avibactam and the combined antibiotics increased 4, 8, and 4-fold, respectively (Fig. 4C). In contrast, in the presence of eravacycline, aztreonam/avibactam and ceftazidime-avibactam alone, the corresponding MICs were increased 32, 1024, and 128-fold, respectively (Fig. 4A). These results indicated that combinations of eravacycline with aztreonam/avibactam and ceftazidime-avibactam retarded the resistance development of Kp43.

To examine whether the collateral sensitivities and the resistance development suppressive effects of the combinations were reproducible on another clinical isolate, we performed a passage assay with a New Delhi metallo-beta-lactamase (NDM)-1-containing K. pneumoniae clinical isolate Kp17. Three parallel repeats were included in the presence and absence of eravacycline. As shown in Fig. S2A in Supplemental File 1, the MIC of eravacycline increased from 0.25 mg/L to 8 mg/L within 6 days and remained stable afterward. We then took a single colony from each of the repeats on day 8 and designated the stains Kp17-E1 to KP17-E3, while those passaged without eravacycline were designated Kp17-C1 to KP17-C3. The full-length *lon* gene was amplified by PCR and analyzed by sequencing. Mutations were identified in the *lon* genes in Kp17-E1, Kp17-E2, and Kp17-E3 at different locations (Fig. S2B in Supplemental File 1), whereas no mutation was found in the *lon* genes of the strains of Kp17-C1-C3. Because Kp17 was resistant to multiple antibiotics used in cloning, including apramycin, we were unable to complement the mutants by introducing a wild-type *lon* gene into the evolved strains.

We next examined the collateral sensitivity of the three evolved eravacycline-resistant mutants. The parental strain Kp17 was highly resistant to ceftazidime-avibactam because avibactam is unable to inhibit NDM-1 (27). However, Kp17 was susceptible to aztreonam/avibactam, which might be due to the resistance of aztreonam to NDM-1 (28). Interestingly, all the evolved eravacycline-resistant strains displayed a 2-fold reduction in the MIC of aztreonam/avibactam (Table S8 in Supplemental File 1). Meanwhile, the evolved aztreonam/avibactam-resistant strains displayed a 2 to 4-fold reduction in the MIC of eravacycline (Table S8 in Supplemental File 1), thus demonstrating reciprocal collateral sensitivity. The combination of eravacycline and aztreonam/avibactam suppressed the resistance development of Kp17 (Fig. S3A and B in Supplemental File 1).

### *In vitro* and *in vivo* antimicrobial effects of eravacycline in combination with aztreonam/avibactam and ceftazidime-avibactam.

In addition to suppression of resistance development, another important objective of combination therapy is to improve antimicrobial efficacies (29). We, thus, performed checkerboard analyses on eravacycline in combination with either aztreonam/avibactam or ceftazidime-avibactam by using Kp43. The combination of eravacycline with aztreonam/avibactam or ceftazidime-avibactam resulted in a fractional inhibitory concentration index (FIC) index of 0.5039, which was close to the criterion of synergy (≤0.5) (30).

**FIG 5:**
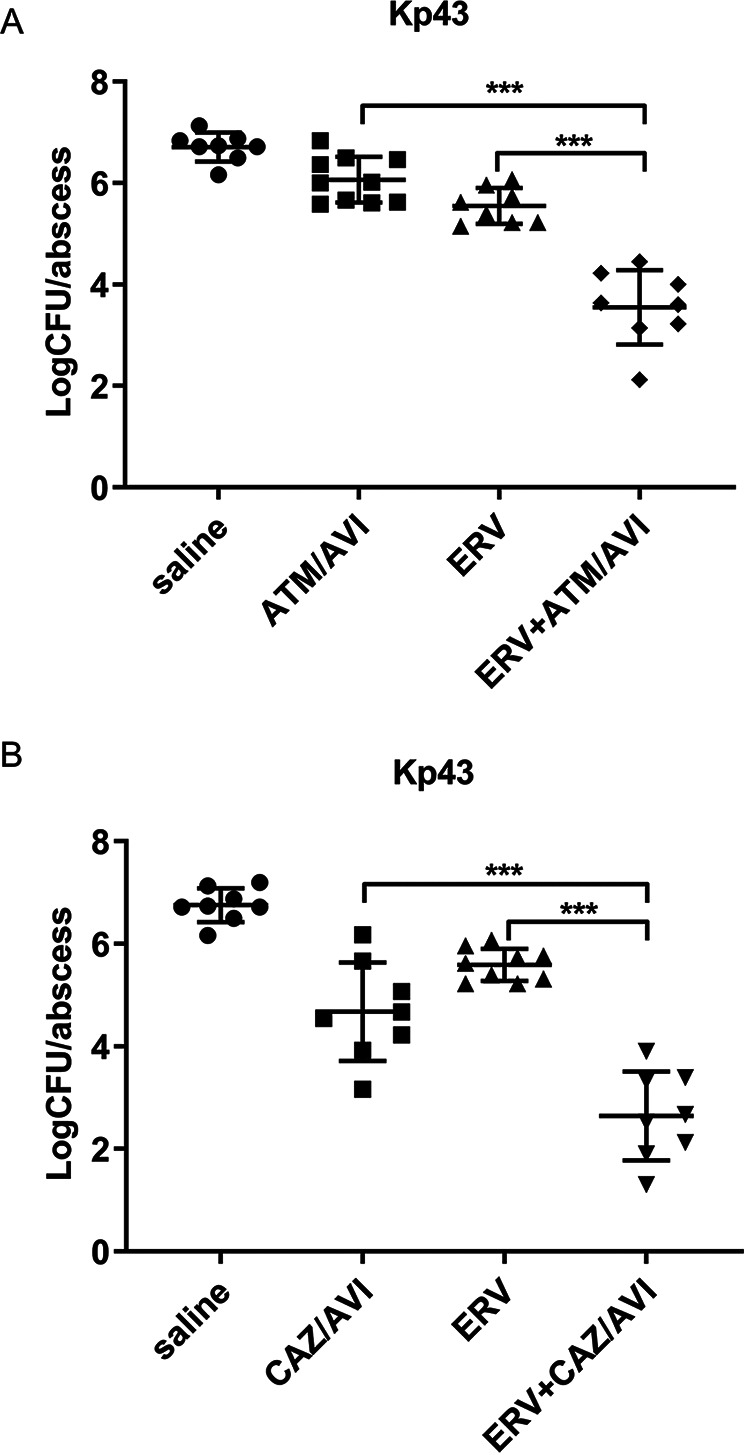
Killing efficacies of eravacycline in combination with aztreonam/avibactam and ceftazidime-avibactam on Kp43 *in vivo*. There were 8 to 10 mice in each group. A total of 2 × 10^7^ CFU of Kp43 was injected subcutaneously into each mouse. Then, each mouse was treated with saline, 0.5 mg/kg eravacycline (ERV), 18 mg/kg aztreonam plus 4.5 mg/kg avibactam (ATM/AVI) (A) or 6 mg/kg ceftazidime plus 1.5 mg/kg avibactam (CAZ/AVI) (B) or the combinations. Twenty hours after the infection, the skin abscesses were excised, and the number of live bacteria was determined by plating. The mean CFU of the bacteria is presented as horizontal lines. ***, *P* < 0.001, by Student's *t* test.

Next, we utilized a mouse cutaneous abscess model to examine the *in vivo* bactericidal activities of the combinations. The dose of each drug was used following previous reports (31, 32). Compared to the saline-treated control group, eravacycline, aztreonam/avibactam, and ceftazidime-avibactam alone reduced the mean bacterial loads by 15, 5, and 120-fold, respectively (Fig. 5A and B). However, the combinations of eravacycline with aztreonam/avibactam and ceftazidime-avibactam reduced the mean bacterial loads by 10^4^-fold and 10^5^-fold, respectively (Fig. 5A and B), indicating synergistic bactericidal effects of the combinations against Kp43 *in vivo*.

## DISCUSSION

The increasing antibiotic resistance demands new antibiotics (33). However, compared to the lengthy process of drug development, the evolution of bacterial resistance is much faster, thus turning the time and prospects against us. Therefore, it is critical to developing strategies to suppress antibiotic resistance development to expand the lifespans of currently used and novel antibiotics.

Eravacycline is a recently approved fluorocycline of the tetracycline class. It is active against a variety of pathogens, including ESBL-producing E. coli, K. pneumoniae, and Acinetobacter spp (34). In this study, we demonstrated that a carbapenem-resistant K. pneumoniae clinical isolate, Kp43, quickly developed resistance to eravacycline. WGS analyses of the resistant mutants revealed mutations that disrupted the *lon* gene or a Tn insertion upstream of the *lon* gene that reduced its expression level. Complementation with a wild-type *lon* gene reduced bacterial resistance to eravacycline. In addition, mutations in the *lon* gene were identified in eravacycline-resistant strains evolved from an NDM-1-containing K. pneumoniae clinical isolate. Previous studies identified mutations in the *lon* gene in tigecycline-resistant K. pneumoniae strains (35, 36), indicating a role of Lon in the resistance to the structure similar to tigecycline and eravacycline.

Lon is a conserved ATP-dependent cytoplasmic serine protease that plays important physiological roles in bacteria (37, 38). It has been demonstrated that Lon directly targets RamA and BamA, which control the expression of the AcrAB-TolC and OqxAB efflux pumps as well as the organization of outer membrane proteins, respectively (39–42). A previous study demonstrated overexpression of MacAB and OqxAB efflux pumps in eravacycline-resistant K. pneumoniae clinical isolates, which might be due to upregulation of *ramA* (18). The expression of *ramA* is repressed by RamR (43). It has been demonstrated that mutations in the *ramR* gene increased tigecycline resistance in K. pneumoniae (36, 43). Our proteomic analyses of membrane proteins revealed an increased amount of AcrAB-TolC in Kp43-E4, which might contribute to eravacycline resistance (44, 45). We proposed that mutation in the *lon* gene might increase the RamA level, which subsequently increased the expression of AcrAB-TolC and thus the resistance to eravacycline and tigecycline. In addition, we found upregulation of OmpA and OmpU in the outer membrane. Overexpression of OmpA and OmpU in Kp43 reduced the MICs of the BLBLI combinations, indicating a possible role of OmpA and OmpU in the influx of the drugs. The accumulation of BamA might promote the assembly of porins on the outer membrane. Besides mutations in the *lon* gene, a frameshift mutation was identified in a DEAD/DEAH box helicase gene on the plasmid pKp43_1. The DEAD/DEAH box helicase might be involved in the processing of rRNA, which might contribute to resistance to eravacycline (46). Further experiments are needed to examine the role of the mutation.

In addition to eravacycline, Kp43 is susceptible to ceftazidime-avibactam and aztreonam/avibactam. Ceftazidime-avibactam was approved for clinical usage by the US Food and Drug Administration (FDA) and the European Medicines Agency (EMA) in 2015 and 2016, respectively. However, ceftazidime-avibactam-resistant K. pneumoniae clinical isolates were reported in 2016 and thereafter (47–49). *In vitro* passage assays demonstrated quick development of ceftazidime-avibactam resistance by a K. pneumoniae clinical isolate (50, 51). In both clinical settings and the laboratory, the resistance was mainly due to amino acid substitution mutations in the β-lactamase genes, which might confer resistance to avibactam (47–51). For aztreonam/avibactam, analyses on laboratory and clinically developed resistant strains revealed point mutations in β-lactamase genes *bla*_PER-4_, *bla*_CMY-16_, etc., and the gene encoding PBP3, the target of aztreonam (52, 53). In addition, mutations in the outer membrane proteins OmpK35 and OmpK36 can increase bacterial resistance to both ceftazidime-avibactam and aztreonam/avibactam (52, 53).

Here, we found that the evolved eravacycline-resistant mutants of Kp43 displayed collateral sensitivity to ceftazidime-avibactam and aztreonam/avibactam. Meanwhile, the evolved ceftazidime-avibactam-resistant strains were more susceptible to eravacycline. However, the evolved aztreonam/avibactam-resistant strains maintained the same level of resistance to eravacycline. The combination of eravacycline with ceftazidime-avibactam or aztreonam/avibactam suppressed the development of resistance to each of the antibiotics and their combinations. An NDM-1-containing K. pneumoniae clinical isolate (Kp17) displayed reciprocal collateral sensitivity between eravacycline and aztreonam/avibactam. The combination of eravacycline and aztreonam/avibactam also suppressed the development of resistance in Kp17. Further studies are warranted to understand the mechanism that leads to the difference in the collateral sensitivities to aztreonam/avibactam of the eravacycline-resistant mutant evolved from Kp43 and Kp17.

The eravacycline-ceftazidime-avibactam and eravacycline-aztreonam/avibactam combinations displayed close to synergy anti-K. pneumoniae effects *in vitro* and synergistic bactericidal effects *in vivo*. Transcriptomic and proteomic studies are needed to understand the bacterial response to the combinations and elucidate the mechanisms of the synergy.

Eravacycline has been approved for the treatment of complicated intra-abdominal infections (cIAIs) in several countries (31, 32). In this study, we evaluated the treatment efficacies of eravacycline in combination with the BLBLIs with a mouse cutaneous abscess model, which is relatively simple with highly reproducible results. In addition, the model is not rapidly lethal and can be used to examine drug efficacies and bacterium-host interactions (54). To mimic the clinical settings, an intra-abdominal infection model is necessary to further evaluate the efficacies of the antibiotic combinations.

The doses of eravacycline, ceftazidime-avibactam, and aztreonam/avibactam for adults are around 4, 29/7.25, and 29/7.25 mg/kg, respectively (7, 31, 32, 55). Based on the Meeh-Rubner equation, the corresponding doses in mice are around 36, 264/66, and 264/66 mg/kg, respectively. In this study, the doses we used were lower than the corresponding human clinical doses. Therefore, the synergistic bacteria-killing effects were indicative. Further evaluation with human corresponding doses and appropriate infection models are needed to verify the effectiveness.

Kp17 produces the metallo-beta-lactamase (MBL) NDM-1, whose activity depends on zinc. However, the *in vivo* free zinc concentration is lower than that in MHB (56, 57). A recent study demonstrated that meropenem is effective against MBL-producing Enterobacteriaceae clinical isolates in zinc-depleted MHB and murine thigh and lung infection models (57). Therefore, ceftazidime-avibactam and carbapenem antibiotics might be effective against Kp17 *in vivo*. The bactericidal effect of eravacycline in combination with carbapenem antibiotics or ceftazidime-avibactam against Kp17 and other MBL-producing K. pneumoniae clinical isolates could be examined in zinc depleted MHB and infection models. Overall, we studied the resistance development of carbapenem-resistant K. pneumoniae clinical isolates against eravacycline. Based on collateral sensitivities, we found that the combinations of eravacycline with ceftazidime-avibactam and aztreonam/avibactam could achieve two objectives, including suppression of the development of resistance and improvement of the treatment efficacies. Another issue that needs to be considered in the combinations is the pharmacokinetics of the drugs, which may be optimized by formulation or delivery systems. Our results demonstrated that combinations of eravacycline with ceftazidime-avibactam and aztreonam/avibactam might be promising therapeutic strategies against infections caused by carbapenem-resistant K. pneumoniae strains.

## MATERIALS AND METHODS

### Bacterial strains, plasmids, primers, and growth conditions.

The bacterial strains, plasmids, and primers used in the study are listed in Table S4 in Supplemental File 1. The K. pneumoniae clinical isolates Kp43 and Kp17 were isolated from blood and sputum culture samples from two patients in Beijing, China. Gene deletion and complementation were performed as previously described (58). Bacteria were cultured in Luria-Bertani (LB) or cation-adjusted Mueller-Hinton (CAMHB) broth at 37°C. Because the pCasKP is a temperature-sensitive plasmid (58), the strains containing the derived pKP or pKP-*lon*_Kp43_ were cultured at 30°C.

For complementation of the *lon* gene, a fragment containing the promoter region and the *lon* coding sequence was amplified by PCR with primers *lon*F and *lon*R using Kp43 chromosomal DNA as the template (Table S4). The PCR product was cloned into the XbaI-NheI sites of the plasmid pKP, resulting in pKP-*lon*_Kp43_ (Table S4 in Supplemental File 1). The plasmid was transferred into the *lon* mutants by electroporation.

### Antimicrobial susceptibility test.

MICs were determined by the 2-fold serial dilution method in cation-adjusted Mueller-Hinton broth (CAMHB) following the Clinical and Laboratory Standards Institute (CLSI) (59). Avibactam (MedChemExpress, China) was used at a fixed concentration of 4 mg/L (60).

### Determination of fractional inhibitory concentration index (FICI).

The fractional inhibitory concentration index was determined by a standard checkerboard broth microdilution method as previously described (61). Briefly, two drugs were 2-fold diluted in CAMHB at different concentrations ranging from 1/256× MIC to 2× MIC and mixed with an equal volume of bacterial suspensions containing approximately 5 × 10^5^ CFU/mL in a 96-well microliter plate. After incubation at 37°C for 20 h, the MIC values were defined as the lowest concentrations of antibiotics with no visible growth of bacteria. FICIs were calculated using the formula: FICI = (MIC of A drug in combination with B drug/MIC of A drug alone) + (MIC of B drug in combination with A drug/MIC of B drug alone). The results were interpreted according to EUCAST (30) as follows: FICI ≤ 0.5: synergy; 0.5 < FICI ≤ 1: additive; 1 < FICI < 2: indifferent; and FICI ≥ 2: antagonistic.

### *In vitro* evolution of eravacycline-resistant strains.

The strains Kp43 and Kp17 were passaged in the presence or absence of eravacycline. Overnight culture of the bacteria was subcultured into fresh CAMHB with increasing concentrations of eravacycline (0.5×, 1×, 2×, and 4×MIC). After 24 h, cells from the highest concentration of antibiotic that allowed growth to a minimum optical density at 600 nm (OD_600_) of 2.0 were inoculated into fresh CAMHB with increased concentrations of eravacycline (e.g., 1×, 2×, 4×, and 8× MIC) for another round of passage. The passaging was repeated for 8 days, and the replicates were streaked to obtain single colonies for MIC determination.

To examine the development of resistance to antibiotic combinations, overnight cultures of the K. pneumoniae strains were diluted 1:100 into fresh CAMHB with increasing concentrations of the combinations of eravacycline with aztreonam/avibactam or ceftazidime-avibactam (0.25×, 0.5×, and 1× MIC of eravacycline, aztreonam/avibactam, or ceftazidime-avibactam) (Table S9 in Supplemental File 1). MICs of the combinations were determined by mixing eravacycline with aztreonam or ceftazidime at a 1:1 ratio in the presence of 4 mg/L avibactam. After 24 h, cells from the highest concentration of antibiotic that allowed the growth to a minimum OD_600_ of 2.0 were inoculated into freshly CAMHB with increasing concentrations of the antibiotics for another round of passage. The passaging was repeated for 8 days, and the replicates were streaked to obtain single colonies for MIC determination.

### Genome sequencing.

Sequencing of the Kp43 genome was performed by GrandOmics (Beijing, China) by Nanopore sequencing using the PromethION platform. Genomes of the four eravacycline-resistant mutant colonies Kp43-E1, Kp43-E2, Kp43-E3, and Kp43-E4 and one control strain passaged in CAMHB (designated Kp43-C1) were sequenced using the MGISEQ2000 platform. Paired-ended sequenced raw reads were filtered using the fastp (v.0.20.0) preprocessor, and then the clean reads were mapped to the reference genome of the parental strain Kp43 using bwa software. Freebayes, snpEff, and bcftools were used for variant calling of single nucleotide polymorphisms (SNPs), small insertions, and deletions (indels).

### RNA isolation and quantitative real-time PCR (qRT–PCR) analysis.

Bacterial overnight cultures were inoculated into fresh LB medium and grown to an OD_600_ of 1.0. Total RNA was extracted and reverse transcribed to cDNA. The cDNA was mixed with the indicated primers (Table S4 in Supplemental File 1) and SYBR premix *Ex Taq* II (Vazyme, Nanjing, China). Then, qRT–PCR was performed in a Step One Plus system (Life Technologies, USA). The DNA-directed RNA polymerase beta chain gene *rpoB* was used as an internal control.

### Eravacycline accumulation assays.

Eravacycline accumulation was determined as previously described with minor modifications (62). Kp43 and the evolved resistant strains were grown to an OD_600_ of 1 in LB. One milliliter of the bacteria was collected by centrifugation and resuspended in 1 mL LB, followed by incubation with 50 mg/L eravacycline for 15 min. The bacteria were collected by centrifugation and washed with 1 mL of an Mg^2+^ buffer (50% methanol, 10 mM Tris–HCl, 0.1 mM MgCl_2_, 0.2% glucose, pH 8). The bacteria were then resuspended in 1 mL of the Mg^2+^ buffer. A 200 μL bacterial suspension was added to each well of a 96-well plate and the fluorescence (excitation at 400 nm and emission at 520 nm) was immediately measured with a Varioskan Flash reader (Thermo Scientific, Netherlands). For pretreatment with the efflux pump inhibitor PAβN, the bacteria were incubated with 50 mg/L PAβN for 1 h in LB before incubation with eravacycline.

### Ceftazidime influx assay.

The ceftazidime influx assay was performed as previously described with modifications (23). Bacteria were grown to an OD_600_ of 0.8 in LB, followed by incubation with 50 mg/L PAβN for 1 h. The cells were washed once and resuspended in the same volume of PBS. Then, half of the cells were lysed by sonication, and the supernatant was collected after centrifugation. The intact cells or the supernatant were incubated with 64 mg/L ceftazidime at room temperature. The OD_260_ was measured with a Varioskan Flash reader (Thermo Scientific, Netherlands) after 1 h in a 96-well plate.

### Analysis of bacterial membrane proteins by liquid chromatography-tandem mass spectrometry (LC-MS/MS).

Bacterial membrane proteins were extracted using a bacterial membrane protein extraction kit (BestBio, Shanghai, China). Briefly, bacteria were grown in LB to OD_600_ of 1. The bacteria were mixed with 10 mM dithiothreitol (DTT), followed by a water bath at 56°C for 1 h. The samples were cooled, mixed with 55 mM iodoacetamide (IAA), and incubated at room temperature in the dark for 45 min. Then, cold acetone was added to the solution and incubated at −20°C for 30 min. Protein samples were collected by centrifugation at 25,000 g at 4°C for 15 min and air dried. The pellets were resolubilized in buffer (8 M urea, 2 M thiourea, 7 mM SDS, 20 mM Tris-HCl [pH 8.0]), and the protein concentrations were determined by bovine serum albumin (BSA) tryptophan assay. The samples were diluted with 0.5 M triethylammonium bicarbonate (TEAB) and digested with a mixture of trypsin enzyme (trypsin enzyme/substrate protein, 1:20) at 37°C for 4 h. Then, the digested peptides were desalted and freeze-dried.

The LC-MS/MS assays were performed by the Beijing Genomics Institute, Beijing, China. The dried peptide samples were reconstituted with mobile phase A (2% ACN, 0.1% FA) and then centrifuged at 20,000 g for 10 min. The supernatant was taken for injection. Separation was performed by a SCIEX eksigent ultra 2D model nanoliter liquid chromatography. The liquid-phase chromatography-separated peptides were passed to an ESI tandem mass spectrometer.

### Ethic statement.

The animal experiments were conducted following the national guidelines on the use of animals in research. The protocol was approved by the Animal Care and Use Committee of Nankai University, College of Life Sciences (permission number: NK-04-2392012).

### Mouse cutaneous abscess model.

The mouse cutaneous abscess infection was performed as previously described (54) with minor modifications. The fur on the back of each female BALB/c mouse (8 weeks old, weighing approximately 20 g) was removed by shaving, followed by the application of chemical depilatories 1 day before the infection. On the following day, each mouse was anesthetized by intraperitoneal injection of 80 μL 7.5% chloral hydrate. Kp43 was grown to an OD_600_ of 1 in LB, washed twice with sterile saline, and resuspended at 4 × 10^8^ CFU/mL. 50 μL of the bacteria were injected into the right side of the dorsum, resulting in 2 × 10^7^ CFU per mouse. Antibiotics or saline (50 μL) was injected subcutaneously into the infected area at 45 min postinfection. After 20 h, the skin abscesses (including all accumulated pus) were excised and homogenized in sterile saline. The bacterial counts were determined by 10-fold serial dilution with saline and plating.

### Data availability.

The genome sequence of Kp43 was deposited in the NCBI database (SRR18724480). The WGS data of the evolved Kp43 mutants were also deposited in the NCBI database (SRR18748512 to SRR18748516). The protein sequences of Kp43 were included in Supplemental File 2.
