# Supplementary material for: Development of Resistance to Eravacycline by Klebsiella pneumoniae and Collateral Sensitivity-Guided Design of Combination Therapies
Source: Microbiol Spectr. 2022 Aug 16;10(5):e01390-22. doi: 10.1128/spectrum.01390-22 (PMC9603973; doi:10.1128/spectrum.01390-22)
Supplement: Supplemental file 1 — Supplemental material. Download spectrum.01390-22-s0001.pdf, PDF file, 1.2 MB [file spectrum.01390-22-s0001.pdf]

Figure. S1

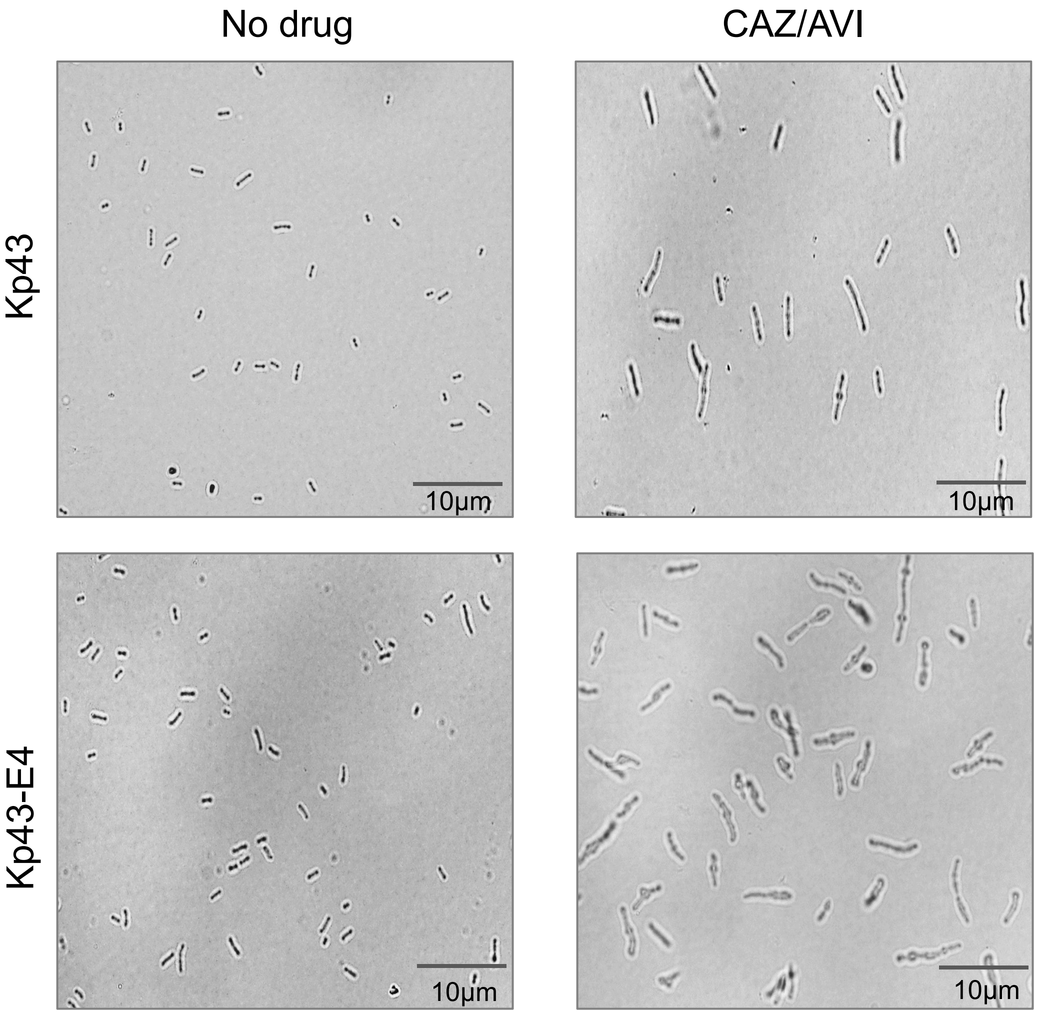

Figure S1. Influx of ceftazidime. Cell morphology in the presence and absence of ceftazidime/avibactam (CAZ/AVI). Bacteria at an OD_{600} of 1.0 were incubated in the presence or absence of 1 mg/L CAZ + 4 mg/L AVI for 60 minutes, followed by microscope observation.

Figure. S2

A

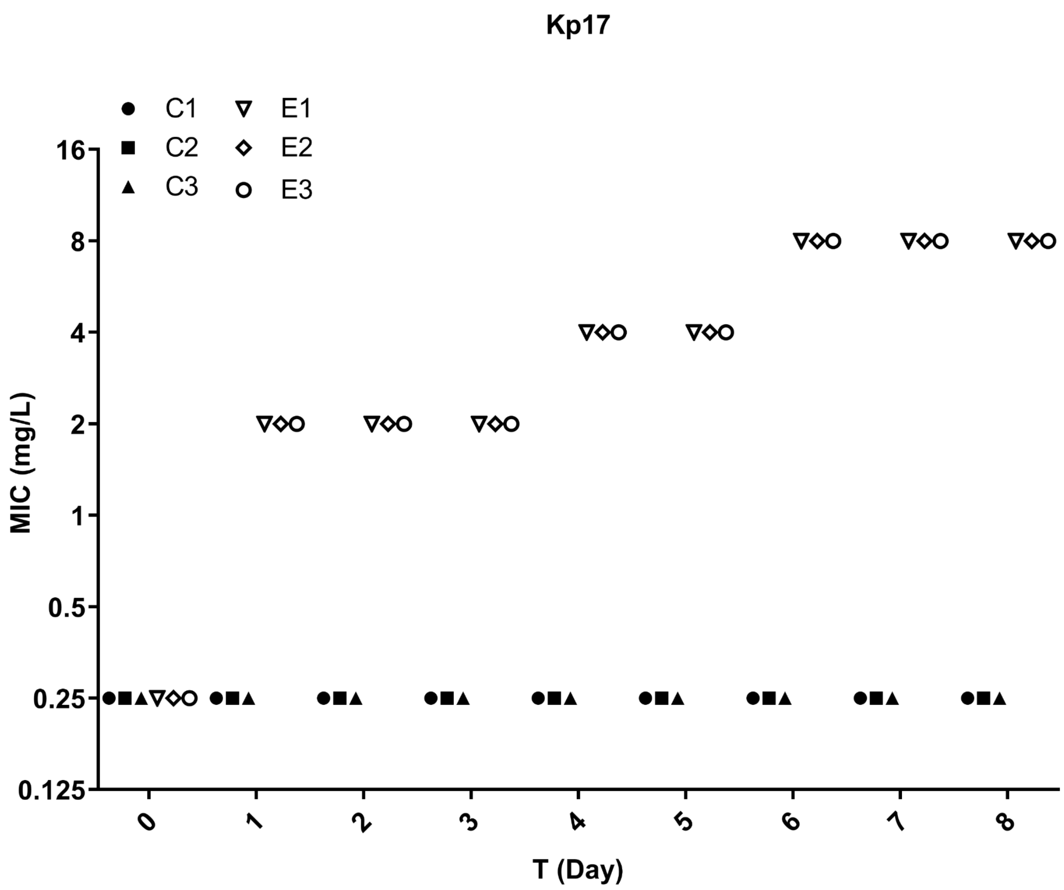

B

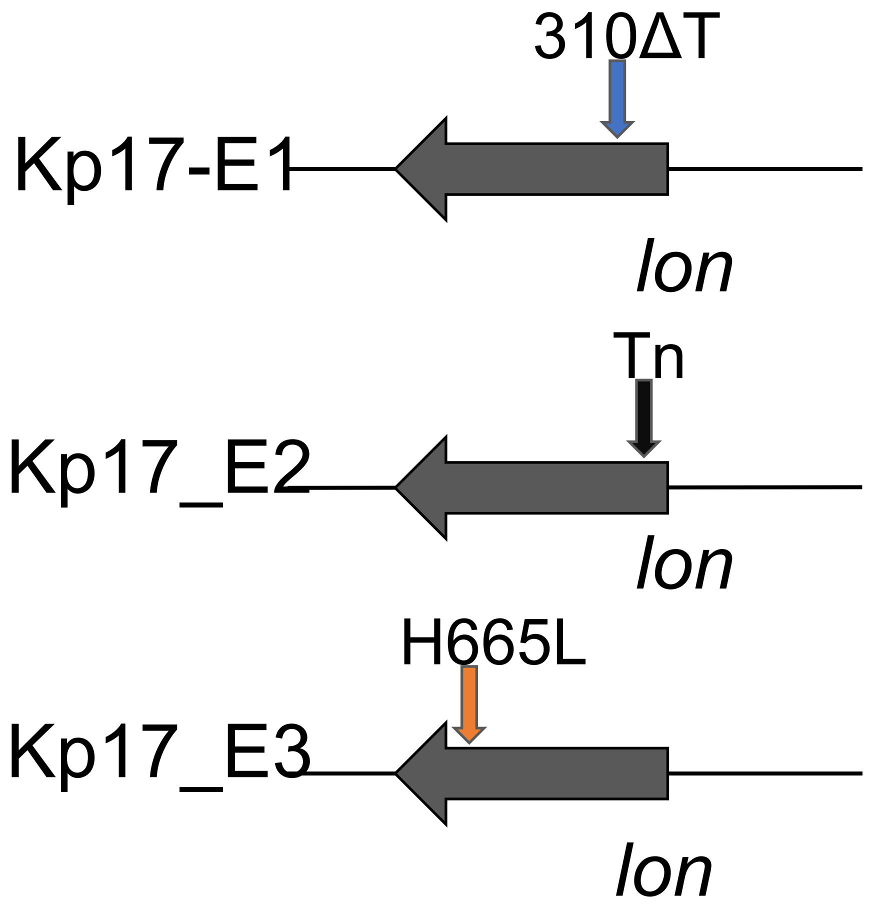

Figure S2. Development of eravacycline resistance by Kp17. (A) Dynamics of stepwise resistance development to eravacycline in Kp17. Three parallel repeats were performed in the passaging with or without eravacycline. (B) Schematic presentation of mutations in the *lon* gene in eravacycline-resistant mutants.

Figure. S3

A

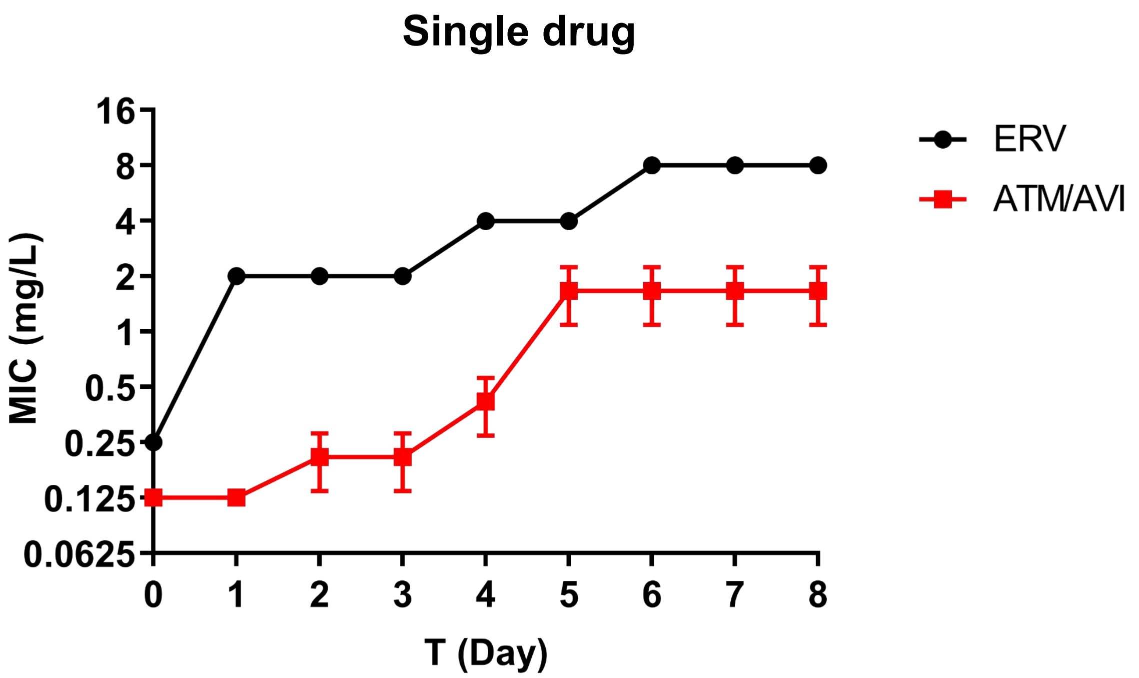

B

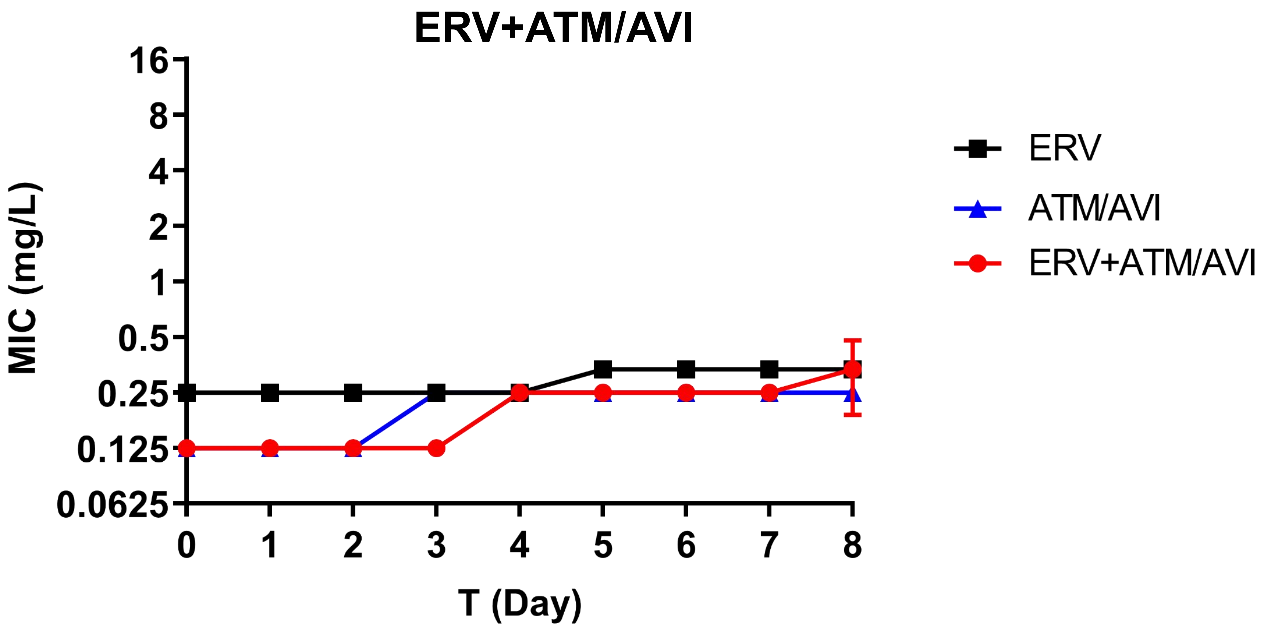

Figure S3. Effects of eravacycline in combination with aztreonam/avibactam on the development of antibiotic resistance in Kp17.(A) Passaging of Kp17 in eravacycline (ERV), aztreonam/avibactam (ATM/AVI) alone. (B) Passaging of Kp17 in ERV+ATM/AVI (1:1). MICs of the individual antibiotics and the corresponding combinations were determined each day. Error bars indicate SEM.

Table S1. General features of the Kp43 genome.

	Chromosome	pKp43-1	pKp43-2	pKp43-3
Size (bp)	5475780	277929	138350	114288
GC content (%)	57.43	46.67	51.72	53.15
ORFs	5248	283	152	127

Table S2. Antibiotic resistance genes in Kp43.

**Location**	**Gene**	**Function**	**Role in resistance**
**Chromosome**	*oqxA. oqxB*	Efflux pump OqxAB coding gene	Quinolone[1]
	fosA	Fosfomycin glutathione transferase	Fosfomycin[2]
	dfrA1	Dihydrofolate reductase (DHFR)	Trimethoprim[3]
	blaSHV-11	A broad-spectrum β-lactamase	β-lactam antibiotics[4]
	sul1	SulP family inorganic anion transporter	trimethoprim/sulfamethoxazole[5]
**pKp43_1**	*catB3*	A class B chloramphenicol acetyltransferase	nonfluorinated phenicols[6]
	aac(6')-Ib-cr	The aminoglycoside 6′-N-acetyltransferase type Ib variant of aminoglycoside acetyltransferases	Fluoroquinolones[7]
	blaOXA-1	A broad-spectrum β-lactamase	β-lactam antibiotics[8]
	aac(6')-Ib-cr	The aminoglycoside 6′-N-acetyltransferase type Ib variant of aminoglycoside acetyltransferases	Fluoroquinolones[7]
	sul1	SulP family inorganic anion transporter	trimethoprim/sulfamethoxazole[5]
	arr-3	Rifampin adenosine diphosphate-ribosyl transferase	Rifampin[9]
**pKp43_2**	*qnrS1*	Quinolone resistance pentapeptide repeat protein QnrS1	Quinolone[1]
	mph(A)	Macrolide 2'-phosphotransferase I	Macrolide[10]
	dfrA1	Dihydrofolate reductase (DHFR)	Trimethoprim[3]
	blaSHV-12	A broad-spectrum β-lactamase	β-lactam antibiotics[4]
	blaLAP-2	A narrow-spectrum β-lactamase	β-lactams[11]
	aac(3)-IId	Aminoglycoside modifying enzymes	Aminoglycoside[12]
	aph(3')-Ia	Aminoglycoside 3'-phosphotransferases type Ia	Aminoglycoside[13]
	tet(A)	Tetracycline efflux pump	Tetracycline[14]
	sul1	SulP family inorganic anion transporter	Trimethoprim/Sulfamethoxazole[5]
**pKp43_3**	*fosA3*	Fosfomycin glutathione transferase	Fosfomycin[15]
	blaTEM-1B	A narrow-spectrum β-lactamase	β-lactams[16]
	blaCTX-M-65	A extended-spectrum β-lactamase	β-lactams[17]
	blaKPC-2	A carbapenemase	Carbapenem[18]
	rmtB	16S rRNA methylase	Aminoglycoside[19]

Table S3. Mutations in the eravacycline-resistant strains.

Strains	Mutation position	Mutation	Gene	Product
Kp43-E1	chromosome	c.C1251A<br>p.Asn417Lys	*lon*	endopeptidase
	pKp43_3	T to A	Intergenic	/
Kp43-E2	chromosome	c.698Δ14bp<br>p.Gln233fs	*lon*	endopeptidase
Kp43-E3	chromosome	c.1339dupG<br>p.Glu447fs	*lon*	endopeptidase
	pKp43_1	c.523dupA<br>p.Met175fs	KP43_05398	DEAD/DEAH box helicase
Kp43-E4	chromosome	c.-10 ins transposase	*lon*	endopeptidase
	chromosome	G to C	Intergenic	/

Table S4. Bacteria, plasmids, primers used in this study.

	Description	Source
Bacteria		
Kp43	*K. pneumoniae* clinical isolates	Lab stock
Kp43-C1	Eravacycline-sensitive *K. pneumoniae* evolved from parental strain Kp43	This study
Kp43-C2	Eravacycline-sensitive *K. pneumoniae* evolved from parental strain Kp43	This study
Kp43-C3	Eravacycline-sensitive *K. pneumoniae* evolved from parental strain Kp43	This study
Kp43-C4	Eravacycline-sensitive *K. pneumoniae* evolved from parental strain Kp43	This study
Kp43-E1	Eravacycline-resistant *K. pneumoniae* evolved from parental strain Kp43	This study
Kp43-E2	Eravacycline-resistant *K. pneumoniae* evolved from parental strain Kp43	This study
Kp43-E3	Eravacycline-resistant *K. pneumoniae* evolved from parental strain Kp43	This study
Kp43-E4	Eravacycline-resistant *K. pneumoniae* evolved from parental strain Kp43	This study
Kp17	*K. pneumoniae* clinical isolates	Lab stock
Kp17-C1	Eravacycline-sensitive *K. pneumoniae* evolved from parental strain Kp17	This study
Kp17-C2	Eravacycline-sensitive *K. pneumoniae* evolved from parental	This study

	strain Kp17	
Kp17-C3	Eravacycline-sensitive *K. pneumoniae* evolved from parental strain Kp17	This study
Kp17-E1	Eravacycline-resistant *K. pneumoniae* evolved from parental strain Kp17	This study
Kp17-E2	Eravacycline-resistant *K. pneumoniae* evolved from parental strain Kp17	This study
Kp17-E3	Eravacycline-resistant *K. pneumoniae* evolved from parental strain Kp17	This study
P4325	*K. pneumoniae* clinical isolates	Lab stock
P4325Δ*lon*	P4325 deletion of *lon*	This study
Plasmids		
pCasKP-apr	Gene expression vector in *K. pneumoniae*, Apr^r	(20)
pKP	pCasKP-apr was digested with *Xba* I and *Nhe* I, the longest fragment purified, ligated using a T4 DNA ligase, Apr^r	This study
pKP-lon_{Kp43}	*lon* gene from Kp43 cloned in pKP, Apr^r	This study
pSGKP-km	Plasmid express sgRNA in *K. pneumoniae*, Km^r	(20)
pSGKP_*lon*	pSGKP-km derivative with *lon* spacer, Km^r	This study
pUCP24	Shuttle vector between *E. Coli* and *P. aeruginosa* pUCP24	
pUCP24NP	Shuttle vector between *E. Coli* and *P. aeruginosa* pUCP24 without promoter, Gm^r	This study
pUCP24NP-lon_{P4325}	*lon* gene from P4325 cloned in pUCP24NP, Gm^r	This study
pUCP24NP-$lon_{Kp43\text{-}E1}$	*lon* gene from Kp43-E1 cloned in pUCP24NP, Gm^r	This study
pUCP24NP-$lon_{Kp43\text{-}E2}$	*lon* gene from Kp43-E2 cloned in pUCP24NP, Gm^r	This study
pUCP24NP-$lon_{Kp43\text{-}E3}$	*lon* gene from Kp43-E3 cloned in pUCP24NP, Gm^r	This study
pUCP24NP-$lon_{Kp43\text{-}E4}$	*lon* gene from Kp43-E4 cloned in pUCP24NP, Gm^r	This study
pUCP24a	The gentamicin resistance cassette of the pUCP24 was replaced with apramycin resistance cassette, Apr^r	This study
pUCP24a-*ompA*	*ompA* gene from Kp43 cloned in pUCP24a, Apr^r	This study
pUCP24a-*ompU*	*ompU* gene from Kp43 cloned in pUCP24a, Apr^r	This study
pUCP24a-*ompAU*	*ompA* and *ompU* gene from Kp43 cloned in pUCP24a, Apr^r	This study

Primer	Sequence 5'-3'	Function	Source
*lon*F	GCTCTAGAGTCCGACAAAGCGAGCGA	Cloning of the *lon* gene in pKP	This study
lonR	CGGCTAGCAGGCTGGCAAGTCCGAAAT		
*lon*F-S	GGGGTACCGTGTGGCGTCGTCGGCTAC	*lon* gene sequencing	This study
*lon*F-R	CCCAAGCTTAGGCTGGCAAGTCCGAAA		
*lon*OEF	GGGGTACCGTCCGACAAAGCGAGCGAAG	For complementation of *lon*	This study
*lon*OER	CCCAAGCTTAGGCTGGCAAGTCCGAAATG		
*ompA*OEF	CGGAATTCCTCCGTAATCGCGCGTCATAATC	Cloning of the *ompA* gene on pUCP24a	This study
*ompA*OER	CGGGATCCTGGCTTCCTTTGCTGAAAATCTG		
*ompU*OEF	CGGGATCCAAAACCCAACAACACGCTATGC	Cloning of the *ompU* gene on pUCP24a	This study
*ompU*OER	CCCAAGCTTGCGATTTGTAGCCACGGTAAGC		
*KPC*OEF	GGGGTACCACCTAGCTCCACCTTCAAAC	Cloning of the *KPC* gene on pUCP24	This study
*KPC*OER	CGGGATCCTTCAGAGCCTTACTGCCCG		
lon-sgRNA-F	TAGTAGGTCGCGTTTGTTCTCATCAGG		
lon-sgRNA-R	AAACCCTGATGAGAACAAACGCGACCT		
lon-up-F	TAGTGGCGACCCTGAACGAG	For deletion of *lon*	This study
lon-up-R	GAACTTCCTCGATTCGTTTCCGGATAAACCACCACATCG		
lon-dn-F	CGATGTGGTGGTTTATCCGGAAACGAATCGAGGAAGTTC		
lon-dn-R	CGCCCTTGTACTTCGTCA		
q*lon*F	AACCGTGGCGTCTATTTTGC	qPCR of *lon*	This study
qlonR	GCTCGCGTTCGTCAATCG		

Table S5. Proteomic analysis: differentially expressed membrane proteins.

Protein ID	Fold Change (Kp43-E4/Kp43)	P value	Regulation	Protein name	Refseq_Description
KPN43_05573	25.36533785	0.00269984	Up	-	YP_007349585.1 hypothetical protein D647_p21106
KPN43_04835	5.966161025	0.00782555	Up	DsbD	WP_004152424.1 protein-disulfide reductase DsbD
KPN43_04589	1.867373828	0.023170993	Up	CstA	WP_002887454.1 MULTISPECIES: carbon starvation protein
KPN43_04172	6.441858469	0.007865857	Up	SbmA	WP_002890204.1 MULTISPECIES: peptide antibiotic transporter SbmA
KPN43_04087	1.636755199	0.033549111	Up	SurA	WP_002891856.1 peptidylprolyl isomerase
KPN43_04047	6.287877041	0.008506462	Up	AcrB	WP_020326861.1 multidrug efflux RND transporter permease subunit
KPN43_04046	4.928649288	0.002061185	Up	AcrA	WP_002892072.1 MULTISPECIES: multidrug efflux RND transporter periplasmic adaptor subunit AcrA
KPN43_04016	3.498053272	0.015960122	Up	YbbP	WP_105177887.1 ABC transporter permease
KPN43_04015	33.65680257	0.036590258	Up	OmpU	WP_004142997.1 MULTISPECIES: porin
KPN43_03994	147.5140991	0.016436416	Up	Lpxl	WP_002892400.1 MULTISPECIES: LpxL/LpxP family Kdo(2)-lipid IV(A) lauroyl/palmitoleoyl acyltransferase
KPN43_03467	3.348222602	0.016092898	Up	PqiB	WP_004179201.1 MULTISPECIES: intermembrane transport protein PqiB
KPN43_03399	4.225910152	0.003622306	Up	GhrA	WP_002898938.1 MULTISPECIES: glyoxylate/hydroxypyruvate reductase GhrA
KPN43_03220	2.917380159	0.02793683	Up	OmpV	WP_002901236.1 MULTISPECIES: MipA/OmpV family protein
KPN43_03160	1.78083335	0.024381578	Up	-	WP_002901627.1 MULTISPECIES: YniB family protein
KPN43_02175	2.234855809	0.046708816	Up	NlpA	WP_002909085.1 MULTISPECIES: MetQ/NlpA family lipoprotein
KPN43_01732	5.58464934	0.038724586	Up	MdtQ	WP_062955093.1 multidrug resistance outer membrane protein MdtQ
KPN43_01635	2.328360971	0.011122293	Up	NuoI	WP_002913152.1 MULTISPECIES: NADH-quinone oxidoreductase subunit NuoI

KPN43_01629	1.849938753	0.005394062	Up	NuoB	WP_002913178.1 MULTISPECIES: NADH-quinone oxidoreductase subunit NuoB
KPN43_01492	7.295683425	0.044963238	Up	PerM	WP_002913806.1 MULTISPECIES: AI-2E family transporter
KPN43_01354	3.661156393	0.031101241	Up	RseA	WP_002914072.1 MULTISPECIES: anti-sigma-E factor RseA
KPN43_01333	1.584628654	0.018224171	Up	BamD	WP_004145664.1 MULTISPECIES: outer membrane protein assembly factor BamD
KPN43_01228	7.148961837	0.033891671	Up	LpxO	WP_002914281.1 MULTISPECIES: lipid A hydroxylase LpxO
KPN43_01084	1.793425234	0.009706163	Up	SdaC	WP_004151070.1 MULTISPECIES: HAAAP family serine/threonine permease
KPN43_00839	1.664233774	0.031934421	Up	MltC	WP_134794262.1 membrane-bound lytic murein transglycosylase MltC
KPN43_00731	2.897378358	0.008965343	Up	TolC	WP_004150921.1 MULTISPECIES: outer membrane channel protein TolC
KPN43_00720	3.518394119	0.030597448	Up	YgiM	WP_002916862.1 MULTISPECIES: SH3 domain-containing protein
KPN43_00615	2.17507483	0.028675202	Up	YraP	WP_002918214.1 MULTISPECIES: divisome-associated lipoprotein YraP
KPN43_00584	2.615156738	0.024657312	Up	FtsH	WP_002918372.1 MULTISPECIES: ATP-dependent zinc metalloprotease FtsH
KPN43_00248	3.513019211	0.023663572	Up	OmpA	WP_086472060.1 OmpA family lipoprotein
KPN43_00244	2.267449644	0.017429767	Up	-	WP_002921927.1 MULTISPECIES: MFS transporter
KPN43_00207	34.32953575	0.008172162	Up	LldD	WP_002922420.1 MULTISPECIES: FMN-dependent L-lactate dehydrogenase LldD
KPN43_01708	0.648869542	0.018928524	Down	FrwC	WP_113851485.1 PTS fructose transporter subunit IIBC
KPN43_01358	0.635137958	0.046727694	Down	LepA	WP_002914069.1 MULTISPECIES: elongation factor 4
KPN43_03506	0.616892561	0.044620677	Down	RpsA	WP_002898162.1 MULTISPECIES: 30S ribosomal protein S1
KPN43_00300	0.586178258	0.045243711	Down	PitA	WP_138975586.1 inorganic phosphate transporter PitA%2C partial
KPN43_04934	0.578918043	0.013618234	Down	LamB	WP_062955053.1 maltoporin
KPN43_01796	0.575997872	0.007022964	Down	Wza	WP_048330978.1 MULTISPECIES: polysaccharide export protein

KPN43_00743	0.548149241	0.010842138	Down	GyrA	WP_002916828.1 MULTISPECIES: DNA topoisomerase IV subunit A
KPN43_04405	0.541929973	0.034026108	Down	FtsA	WP_002888625.1 MULTISPECIES: cell division protein FtsA
KPN43_03522	0.527899358	0.010982489	Down	FtsK	WP_048270395.1 DNA translocase FtsK
KPN43_03154	0.475892737	0.001733384	Down	OmpW	WP_002901634.1 MULTISPECIES: outer membrane protein OmpW
KPN43_04938	0.434583117	0.004257148	Down	MalG	WP_002884725.1 MULTISPECIES: maltose ABC transporter permease MalG
KPN43_03641	0.427088135	0.011359792	Down	GlnH	WP_002895839.1 MULTISPECIES: glutamine ABC transporter substrate-binding protein GlnH
KPN43_03729	0.362565949	0.020502958	Down	TolA	-
KPN43_00553	0.362452349	0.032839012	Down	ArcB	WP_153938016.1 aerobic respiration two-component sensor histidine kinase ArcB
KPN43_05209	0.340911481	0.030396433	Down	RbsA	WP_002882529.1 MULTISPECIES: ribose ABC transporter ATP-binding protein RbsA
KPN43_04051	0.33861662	0.009448952	Down	-	WP_002892026.1 MULTISPECIES: YlaC family protein
KPN43_03708	0.319637351	0.041139389	Down	ModA	WP_004151692.1 molybdate ABC transporter substrate-binding protein
KPN43_01928	0.313427307	4.42E-05	Down	HisJ	WP_135658896.1 cystine ABC transporter substrate-binding protein
KPN43_03347	0.283730203	0.010908419	Down	CirA	WP_004150817.1 MULTISPECIES: TonB-dependent siderophore receptor
KPN43_00884	0.248465122	0.000128827	Down	MscS	WP_002916504.1 MULTISPECIES: small-conductance mechanosensitive channel MscS
KPN43_05644	0.238295148	0.031138796	Down	SilP	WP_135713579.1 Ag(+)-translocating P-type ATPase SilP
KPN43_03568	0.232511253	0.00148444	Down	YbjL	WP_004179131.1 MULTISPECIES: aspartate:alanine antiporter
KPN43_05529	0.227852264	0.010443069	Down	-	WP_001334766.1 MULTISPECIES: oxacillin-hydrolyzing class D beta-lactamase OXA-1
KPN43_01361	0.202613323	0.013905089	Down	Era	WP_002914063.1 MULTISPECIES: GTPase Era
KPN43_00379	0.180092001	0.014695041	Down	FeoB	WP_002920508.1 MULTISPECIES: Fe(2+) transporter permease subunit FeoB
KPN43_01835	0.166411364	0.019374176	Down	PlaP	WP_062955129.1 putrescine/proton symporter PlaP

KPN43_02516	0.0566874	0.016248515	Down	-	WP_004151245.1 MULTISPECIES: SDR family oxidoreductase
KPN43_01061	0.562793354	0.01617177	Down	-	WP_004142890.1 YgdI/YgdR family lipoprotein
KPN43_03408	0.567827609	0.021078452	Down	EfeO	WP_004157740.1 MULTISPECIES: iron uptake system protein EfeO

Table S6. KPC gene mutations in the ceftazidime/avibactam and aztreonam/avibactam-resistant strains.

Strains	Gene	Mutation	Mutation in protein
Kp43-CA1	*KPC*	C725T	T242M
Kp43-CA2	*KPC*	C725T	T242M
Kp43-CA3	*KPC*	C518T	P173L
Kp43-CA4	*KPC*	G511A	A171T
Kp43-AA1	*KPC*	T310A	W104R
Kp43-AA2	*KPC*	T310A	W104R
Kp43-AA3	*KPC*	T310A	W104R
Kp43-AA4	*KPC*	C751G	P251A

Table S7. MICs (mg/L) of indicated *K. pneumoniae* strains.

Strains	CAZ/AVI	ATM/AVI
P4325/pUCP24	0.125	0.03125
P4325/pUCP24-$KPC_{\text{Kp43-CA1}}$	0.125	0.0625
P4325/pUCP24-$KPC_{\text{Kp43-CA2}}$	0.125	0.03125
P4325/pUCP24-$KPC_{\text{Kp43-CA3}}$	0.125	0.03125
P4325/pUCP24-$KPC_{\text{Kp43-CA4}}$	0.125	0.03125
P4325/pUCP24-$KPC_{\text{Kp43-AA1}}$	0.125	0.0625
P4325/pUCP24-$KPC_{\text{Kp43-AA2}}$	0.125	0.03125
P4325/pUCP24-$KPC_{\text{Kp43-AA3}}$	0.125	0.03125
P4325/pUCP24-$KPC_{\text{Kp43-AA4}}$	0.125	0.03125

ATM: Aztreonam; CAZ: Ceftazidime; AVI: Avibactam;

Table S8. MICs (mg/L) of indicated *K. pneumoniae* strains.

Strains	ATM/AVI	CAZ/AVI	ERV
Kp17	0.125	>64	0.25
Kp17-C1	0.125	>64	0.25
Kp17-E1	0.0625	>64	8
Kp17-E2	0.0625	>64	8
Kp17-E3	0.0625	>64	8
KP17-AA1	1	ND	0.125
KP17-AA2	2	ND	0.0625
KP17-AA3	2	ND	0.125

ATM: Aztreonam; CAZ: Ceftazidime; AVI: Avibactam; AA: Aztreonam/Avibactam resistant strains from Kp17; ND: Not determined.

References

[1] Zaki MES, El Salam MA, Faried OA. Study of Plasmid Mediated Quinolone Resistance in *Escherichia coli* from Nosocomial Urinary Infections. Infect Disord Drug Targets. 2020 May 20. doi: 10.2174/1871526520666200520112319. Epub ahead of print. PMID: 32433009.

[2] Elliott ZS, Barry KE, Cox HL, et al. The Role of fosA in Challenges with Fosfomycin Susceptibility Testing of Multispecies *Klebsiella pneumoniae* Carbapenemase-Producing Clinical

Isolates. J Clin Microbiol. 2019;57(10):e00634-19. Published 2019 Sep 24. doi:10.1128/JCM.00634-19

[3] Lombardo MN, G-Dayanandan N, Wright DL, Anderson AC. Crystal Structures of Trimethoprim-Resistant DfrA1 Rationalize Potent Inhibition by Propargyl-Linked Antifolates. ACS Infect Dis. 2016 Feb 12;2(2):149-56. doi: 10.1021/acsinfecdis.5b00129. Epub 2016 Jan 4. PMID: 27624966; PMCID: PMC5108240.

[4] Lee M, Choi TJ. Species Transferability of *Klebsiella pneumoniae* Carbapenemase-2 Isolated from a High-Risk Clone of *Escherichia coli* ST410. J Microbiol Biotechnol. 2020 Jul 28;30(7):974-981. doi: 10.4014/jmb.1912.12049. PMID: 32522962.

[5] Chung HS, Kim K, Hong SS, Hong SG, Lee K, Chong Y. The sul1 gene in *Stenotrophomonas maltophilia* with high-level resistance to trimethoprim/sulfamethoxazole. Ann Lab Med. 2015;35(2):246-249. doi:10.3343/alm.2015.35.2.246

[6] Kadlec K, Schwarz S. Antimicrobial Resistance in *Bordetella bronchiseptica*. Microbiol Spectr. 2018 Jul;6(4). doi: 10.1128/microbiolspec.ARBA-0024-2017. PMID: 30027886.

[7] Silva SMD, Ramos BA, Lima AVA, et al. First report of the aac(6')-Ib-cr gene in *Providencia stuartii* isolates in Brazil. *Rev Soc Bras Med Trop*. 2020;54:e20190524. Published 2020 Nov 13. doi:10.1590/0037-8682-0524-2019

[8] Sugumar M, Kumar KM, Manoharan A, Anbarasu A, Ramaiah S. Detection of OXA-1 β-lactamase gene of *Klebsiella pneumoniae* from blood stream infections (BSI) by conventional PCR and in-silico analysis to understand the mechanism of OXA mediated resistance. PLoS One. 2014 Mar 19;9(3):e91800. doi: 10.1371/journal.pone.0091800. PMID: 24647004; PMCID: PMC3960141.

[9] Royer G, Fourreau F, Gomart C, Maurand A, Hacquin B, Ducellier D, Cizeau F, Lo S, Cordonnier-Jourdin C, Mercier-Darty M, Decousser JW. Outbreak of an Uncommon Rifampin-resistant blaNDM-1*Citrobacter amalonaticus* Strain in a Digestive Rehabilitation Center: The Putative Role of Rifaximin. Clin Infect Dis. 2020 Aug 22;71(5):1331-1333. doi: 10.1093/cid/ciz1172. PMID: 31811283.

[10] Salah M, Shtayeh I, Ghneim R, Al-Qass R, Sabateen A, Marzouqa H, Hindiyeh M. Evaluation of Shigella Species Azithromycin CLSI Epidemiological Cutoff Values and Macrolide Resistance Genes. J Clin Microbiol. 2019 Mar 28;57(4):e01422-18. doi: 10.1128/JCM.01422-18. PMID: 30700507; PMCID: PMC6440796.

[11] Kocsis B, Kocsis E, Fontana R, Cornaglia G, Mazzariol A. Identification of blaLAP-$_2$ and qnrS1 genes in the internationally successful *Klebsiella pneumoniae* ST147 clone. J Med Microbiol. 2013 Feb;62(Pt 2):269-273. doi: 10.1099/jmm.0.050542-0. Epub 2012 Nov 1. PMID: 23118475.

[12] Hoard Amparo,Montaña Sabrina,Moriano Alessandro et al. Genomic Analysis of two NDM-1 *Providencia stuartii* Strains Recovered from a Single Patient.[J] .Curr Microbiol, 2020, 77: 4029-4036

[13] Siregar JJ, Miroshnikov K, Mobashery S. Purification, characterization, and investigation of the mechanism of aminoglycoside 3'-phosphotransferase type Ia. Biochemistry. 1995 Oct 3;34(39):12681-8. doi: 10.1021/bi00039a026. PMID: 7548020.

[14] Lynch C, Hawkins K, Lynch H, Egan J, Bolton D, Coffey A, Lucey B. Investigation of molecular mechanisms underlying tetracycline resistance in *thermophilic Campylobacter* spp. suggests that previous reports of *tet*(A)-mediated resistance in these bacteria are premature. Gut Pathog. 2019 Nov 9;11:56. doi: 10.1186/s13099-019-0338-1. PMID: 31728161; PMCID: PMC6842541.

[15] Ito R, Pacey MP, Mettus RT, Sluis-Cremer N, Doi Y. Origin of the plasmid-mediated fosfomycin resistance gene fosA3. J Antimicrob Chemother. 2018 Feb 1;73(2):373-376. doi: 10.1093/jac/dkx389. PMID: 29106538; PMCID: PMC5890757.

[16] Peng Z, Li X, Hu Z, Li Z, Lv Y, Lei M, Wu B, Chen H, Wang X. Characteristics of Carbapenem-Resistant and Colistin-Resistant *Escherichia coli* Co-Producing NDM-1 and MCR-1 from Pig Farms in China. Microorganisms. 2019 Oct 23;7(11):482. doi: 10.3390/microorganisms7110482. PMID: 31652858; PMCID: PMC6920953.

[17] Zong Z, Yu R, Wang X, Lü X. blaCTX-M-65 is carried by a Tn1722-like element on an IncN conjugative plasmid of ST131 *Escherichia coli*. J Med Microbiol. 2011 Apr;60(Pt 4):435-441. doi: 10.1099/jmm.0.026997-0. Epub 2010 Dec 16. PMID: 21163826.

[18] Dong N, Lin D, Zhang R, Chan EW, Chen S. Carriage of blaKPC-2 by a virulence plasmid in hypervirulent *Klebsiella pneumoniae*. J Antimicrob Chemother. 2018 Dec 1;73(12):3317-3321. doi: 10.1093/jac/dky358. PMID: 30239821.

[19] Uchida Hiroki,Tada Tatsuya,Tohya Mari et al. Emergence in Japan of an isolate of *Klebsiella pneumoniae* co-harbouring bla and rmtB.[J] .J Glob Antimicrob Resist, 2019, 17: 157-159.

[20] Wang, Y., Wang, S., Chen, W., Song, L., Zhang, Y., Shen, Z., Yu, F., Li, M., and Ji, Q. (2018). CRISPR-Cas9 and CRISPR-Assisted Cytidine Deaminase Enable Precise and Efficient Genome Editing in *Klebsiella pneumoniae*. *Appl Environ Microbiol* 84:e01834. doi: 10.1128/AEM.01834-18.

Table S9. Concentrations (mg/L) of antibiotics in the passaging experiment.

Concentrations of eravacycline in the passaging experiment for Kp43

	Parallel 1	Parallel 2	Parallel 3	Parallel 4
Day1	1,2,4,8	1,2,4,8	1,2,4,8	1,2,4,8
Day2	1,2,4,8	1,2,4,8	1,2,4,8	1,2,4,8
Day3	4,8,16	4,8,16	4,8,16	4,8,16
Day4	8,16,32,64	8,16,32,64	8,16,32,64	8,16,32,64
Day5	16,32,64	16,32,64	16,32,64,128	16,32,64
Day6	32,64	32,64	32,64	32,64
Day7	32,64	32,64	32,64	32,64
Day8	32,64	32,64	32,64	32,64

Concentrations of antibiotics in the passaging experiment for Kp43

	ERV		ATM/AVI				ERV+ATM/AVI		CAZ/AVI				ERV+CAZ/AVI	
	Parallel 1	Parallel 2	Parallel 1	Parallel 2	Parallel 3	Parallel 4	Parallel 1	Parallel 2	Parallel 1	Parallel 2	Parallel 3	Parallel 4	Parallel 1	Parallel 2
Day1	1,2	1,2	0.25,0.5,1	0.25,0.5,1	0.25,0.5,1	0.25,0.5,1	0.125,0.25,0.5	0.125,0.25,0.5	1,2,4	1,2,4	1,2,4	1,2,4	0.5,1,2	0.5,1,2
Day2	2,4	2,4	0.5,1,2	0.5,1,2	0.5,1,2	0.5,1,2	0.125,0.25,0.5	0.25,0.5,1	2,4,8	2,4,8	2,4,8	2,4,8	0.5,1,2	0.5,1,2
Day3	4,8	4,8	1,2,4	1,2,4	2,4,8	2,4,8	0.25,0.5,1	0.25,0.5,1	8,16,32	8,16,32	8,16,32	8,16,32	0.5,1,2	1,2,4
Day4	8,16	8,16	2,4,8	2,4,8	4,8,16	4,8,16	0.5,1,2	0.5,1,2	16,32,64	16,32,64	16,32,64	16,32,64	1,2,4	4,8,16

Day5	16,32	16,32	4,8,16	4,8,16	8,16,32	8,16,32	1,2,4	0.5,1,2	16,32,64	32,64,128	32,64,128	32,64,128	2,4,8	2,4,8
Day6	32,64	32,64	8,16,32	8,16,32	16,32,64	16,32,64	2,4,8	1,2,4	32,64,128	64,128,256	32,64,128	32,64,128	4,8,16	4,8,16
Day7	32,64	32,64	32,64	32,64	64,128,256	64,128,256	2,4,8	1,2,4	64,128	128,256	32,64,128	32,64,128	4,8,16	4,8,16
Day8	32,64	32,64	64,128	64,128	64,128,256	64,128,256	2,4,8	2,4,8	64,128,256	64,128,256	32,64,128	32,64,128	4,8	4,8

Concentrations of antibiotics in the passaging experiment for Kp17

	ERV			ATM/AVI			ERV+ATM/AVI		
	Parallel 1	Parallel 2	Parallel 3	Parallel 1	Parallel 2	Parallel 3	Parallel 1	Parallel 2	Parallel 3
Day1	0.125,0.25	0.125,0.25	0.125,0.25	0.0625,0.125	0.0625,0.125	0.0625,0.125	0.0625,0.125	0.0625,0.125	0.0625,0.125
Day2	0.25,0.5	0.25,0.5	0.25,0.5	0.03125,0.0625	0.03125,0.0625	0.03125,0.0625	0.03125,0.0625	0.03125,0.0625	0.03125,0.0625
Day3	0.5,1	0.5,1	0.5,1	0.0625,0.125,0.25	0.03125,0.0625,0.125	0.0625,0.125,0.25	0.03125,0.0625,0.125	0.03125,0.0625,0.125	0.03125,0.0625,0.125
Day4	1,2	1,2	1,2	0.0625,0.125,0.25	0.0625,0.125,0.25	0.0625,0.125,0.25	0.03125,0.0625,0.125	0.03125,0.0625,0.125	0.03125,0.0625,0.125
Day5	2,4	2,4	2,4	0.0625,0.125,0.25	0.0625,0.125,0.25	0.0625,0.125,0.25	0.0625,0.125	0.0625,0.125	0.0625,0.125
Day6	4,8	4,8	4,8	0.125,0.25,0.5	0.25,0.5,1	0.25,0.5,1	0.0625,0.125	0.0625,0.125	0.0625,0.125
Day7	4,8	4,8	4,8	0.125,0.25,0.5	1,2,4	0.125,0.25,0.5	0.0625,0.125,0.25	0.0625,0.125,0.25	0.0625,0.125,0.25
Day8	4,8	4,8	4,8	0.5,1,2	4,8,16	0.5,1,2	0.0625,0.125,0.25	0.0625,0.125,0.25	0.0625,0.125,0.25

ERV, eravacycline; ATM, aztreonam; CAZ, ceftazidime; AVI, avibactam

AVI: 4 mg/L, ERV:ATM/AVI=1:1, ERV:CAZ/AVI=1:1
